# Systematic Analysis of Absorbed Anti-Inflammatory Constituents and Metabolites of *Sarcandra glabra* in Rat Plasma Using Ultra-High-Pressure Liquid Chromatography Coupled with Linear Trap Quadrupole Orbitrap Mass Spectrometry

**DOI:** 10.1371/journal.pone.0150063

**Published:** 2016-03-14

**Authors:** Xiong Li, Jin Zhao, Jianxing Liu, Geng Li, Ya Zhao, Xing Zeng

**Affiliations:** 1 Second Affiliated Hospital, Guangzhou University of Chinese Medicine, Guangzhou, China; 2 Zhongshan City People's Hospital, Zhongshan, China; 3 School of Chinese Pharmaceutical Science, Guangzhou University of Chinese Medicine, Guangzhou, China; 4 The postdoctoral research station, Guangzhou University of Chinese Medicine, Guangzhou, China; Jadavpur University, INDIA

## Abstract

Ultra-high-pressure liquid chromatography (UHPLC) was coupled with linear ion trap quadrupole Orbitrap mass spectrometry (LTQ-Orbitrap) and was used for the first time to systematically analyze the absorbed components and metabolites in rat plasma after oral administration of the water extract of *Sarcandra glabra*. This extract is a well-known Chinese herbal medicine for the treatment of inflammation and immunity related diseases. The anti-inflammatory activities of the absorbed components were evaluated by measuring nitric oxide (NO) production and proinflammatory genes expression in lipopolysaccharide (LPS)-stimulated murine RAW 264.7 macrophages. As a result, 54 components in *Sarcandra glabra* were detected in dosed rat plasma, and 36 of them were positively identified. Moreover, 23 metabolites were characterized and their originations were traced. Furthermore, 20 of the 24 studied components showed anti-inflammatory activities. These results provide evidence that this method efficiency detected constituents in plasma based on the anti-inflammatory mechanism of multiple components and would be a useful technique for screening multiple targets for natural medicine research.

## Introduction

Traditional Chinese Medicine (TCM) has been clinically used for thousands of years in China, and it is comprised of multiple components, which is in contrast to Western medicine, where single active chemicals are used. The components of TCM formulations are considered responsible for their therapeutic effects by exerting their synergistic effects on multiple targets and levels. However, only the absorbed constituents and their metabolites ultimately reach these biological targets and, therefore, should be considered the main factors that mediate the health effects of TCM preparations. Therefore, the systematic analysis and identification of the absorbed components of TCM preparations and their metabolites is critical to further understanding the underlying pharmacological mechanisms and enhancing the clinical application of TCM. However, there are two major obstacles to the further pharmacological investigations of TCM formulations. Firstly, the detection and structural characterization of chemical constituents contained in herbal prescriptions are often challenging owing to the complex methods required for identifying, isolating and preparing those components. Secondly, the analysis and profiling of the absorbed constituents of TCMs and their metabolites are difficult because of endogenous matrix interference, as well as the molecular diversity of metabolic pathways. The introduction of the new analytical technique, especially the coupling of ultra-pressure liquid chromatography (UPLC) and mass spectrometry (MS) has provided a powerful tool for the structural characterization of components of natural-based medicines and biological media [1], However, it is still a challenge to conduct a thorough identification of multiple metabolites within a complex, unpurified biological medium.

*Sarcandra glabra* (Thunb) Nakai (family Chloranthaceae) is a a medicinal herb that mainly grows in the south of China. The whole *S*. *glabra* plant and its water extract have been listed in the Chinese Pharmacopoeia, while its single prescription preparations, such as Zhongjiefeng tablets and Xiekang capsules, are mainly used to treat inflammation and immune-related diseases such as acute respiratory infections, thrombocytopenia, pneumonia, cellulitis, appendicitis, shigellosis, psoriasis, and malignancies [2]. In this present study, for the first time, we have developed a rapid analytical method based on a UHPLC system coupled to a photodiode array detection (PDA) and a linear ion trap high-resolution mass spectrometer (LTQ-Orbitrap XL) for qualitative identification of the constituents of *S*. *glabra* and its related preparations [3]. Furthermore, a rapid and reliable high-performance liquid chromatography- electrospray ionization-tandem mass spectrometry (HPLC-ESI-MS/MS) method was further established to quantify 17 main compounds isolated from *S*. *glabra* and its preparations [4]. However, little is known about the specific ingredients that are absorbed into the blood or their final *in vivo* fate following oral administration. This limit understanding is a major obstacle to the performance of further pharmacological mechanistic studies and, therefore, it is necessary to establish an analytical method to profile the metabolites of *S*. *glabra* following their absorption.

The aims of this study was to develop a full-scale strategy for the systematic screening and identification of the absorbed anti-inflammatory components of *S*. *glabra* as well as its metabolites. We based our novel procedure on a UHPLC system combined with an LTQ Orbitrap hybrid mass spectrometer method to comprehensively elucidate of the metabolism of *S*. *glabra* and its potential active metabolites.

## Materials and Methods

### Chemicals and Materials

The dried whole plants of *S*. *glabra* (Batch number 121009391), originating from the Sichuan Province in China, were purchased from Kangmei Pharmaceutical Co. Ltd, China.

Quinic acid (**1**), shikimic acid (**3**), fraxin (**22**), eculetin (**24**), fraxidin (**31**), taxifolin (**41**), and quercitrin (**67**) were purchased from Weikeqi Standards Corporation, Sichuan, China. *P*-coumaric acid (**98**), isoferulic acid (**135**), and ferulic acid (**136**) were purchased from Shanghai Pure One Biotechnology, China. Phlorizin (**61**) was provided by Dr Zhao. The 4, 5-dihydroxy-7-rhamnyl-2H-chromen-2-one (**44**), quercetin-3-*O*-*β-*D-glucuronide (**57**), and 5-hydroxy-7, 8-dimethoxy-flavanone (**88**) were isolated from the whole plants of *S*. *glabra*, while isofraxidin-7-O-sulfate (**100**), and isofraxidin-7-O-α-D-glucuronide (**101)** were isolated from the urine collected following oral administration of isofraxidin to the author. Compounds **57**, **88**, **100** and **101** were further identified using MS and nuclear magnetic resonance (NMR) techniques (see S1 File), and their purities were not less than 90% using an UHPLC analysis. Other standards were also isolated from *S*. *glabra* as previous described [3, 5–8].

The RAW264.7 mouse macrophages used were purchased from the American Type Culture Collection (ATCC, Rockville, MD, USA). The high-glucose Dulbecco’s modified Eagle’s medium (DMEM) and fetal bovine serum (FBS) were purchased from Gibco BRL (NY, USA). The lipopolysaccharide (LPS), dimethylsulfoxide (DMSO) and 3-(4, 5-dimethyl-thiazol-2-yl)-2, 5-diphenyl tetrazolium bromide (MTT) were purchased from Sigma-Aldrich Chemical Corp. (St. Louis, MO, USA). The nitrite assay (nitric oxide, NO) detection kit was purchased from Beyotime Institute of Biotechnology (Jiangsu, China). The tumor necrosis factor (TNF)-α, interleukin (IL)-6 and IL-1β primers, and TRIZOL reagent were purchased from Invitrogen (Carlsbad, CA, USA). The Hypoderm Molecular Biology Grade Water and RevertAid First Strand cDNA Synthesis kit were purchased from Thermo Scientific while the Fast Start Universal SYBR Green master mix (Rox) was purchased from Roche Applied Science.

### Preparation of the *S*. *glabra* extract

The dried *S*. *glabra* plant material (510 g) was extracted by refluxing at 100°C with 4 L water for 1 h, the aqueous solution was filtered and concentrated to 170 mL, and then sealed and stored at temperature below -20°C until it was used or analyzed further. To prepare the analytical sample, the *S*. *glabra* water extract (3 g/mL) was diluted to the a concentration of 0.1 g/mL with 30% methanol, and the solution was filtered through a 0.22-μm nylon membrane filter prior to the LC/MS analysis.

### Animal study and Plasma sample preparation

Thirty-two male Sprague-Dawley rats (weighing 275 ± 25 g) were purchased from Guangdong Medical Laboratory Animal Center (Guangzhou, China) and maintained in air-conditioned animal quarters with alternating 12-h light/dark cycles at a room temperature of 23 ± 1°C and a relative humidity of 60% ± 10% for 1 week prior to the experiments. Commercial rat chow and water were provided *ad libitum*. The animal studies were approved by the Review Committee of Animal Care and Use at the Guangdong Provincial Hospital of TCM (No.2013012). The rats were divided into 10 groups: group A, *S*. *glabra* extract group (3 g/100 g) for dosed rat plasma, n = 5; group B_1_, control group (distilled water) for blank rat plasma, n = 3; group B_2_, control group (0.5% CMCNa) for blank rat plasma, n = 3; group C, chlorogenic acid (3 mg/100 g) for dosed rat plasma, n = 3, group D, eleutheroside B_1_ (3 mg/100 g) for dosed rat plasma, n = 3, group E, isofraxidin (0.75 mg/100 g) for dosed rat plasma, n = 3, group F, astilbin (3 mg/100 g) for dosed rat plasma, n = 3, group G, rosmarinic acid-4-*O*-*β-*D-glucoside (3 mg/100 g) for dosed rat plasma, n = 3, group H, quercetin- 3-*O*-*β-*D-glucuronide (3 mg/100 g) for dosed rat plasma, n = 3, and group I, rosmarinic acid (3 mg/100 g) for dosed rat plasma, n = 3. All the animals were fasted overnight (16 h), and had free access to water throughout the experiment. Then, 1.5 hours after the first oral administration, the rats were gavaged and 30 minutes after the second dose, the rats were anesthetized with 10% chloral hydrate by intraperitoneal injection (0.3 mL/100 g). Then, blood samples were collected via the inferior vena of the abdominal aorta, stored at 4°C for 30 min, and then centrifuged at 3000 rpm for 10 minutes to obtain plasma samples, which were stored at −80°C until they were analyzed.

The plasma samples for each group were mixed to eliminate individual variability, and then, 4 volumes of methanol were added to 1 mL of the mixed rat plasma. The tube of plasma was vortexed for 120 s, and then, the precipitated protein was removed by centrifugation at 13,000 rpm for 10 min. The supernatant was transferred to another tube and evaporated to dryness using nitrogen gas at 37°C_._ The residue was dissolved in 200 μL methanol-water with 0.1% formic acid (4:6, *v/v*), filtered through a 0.22 μm nylon filte, and then transferred into a disposable glass auto-sampler vials. Finally, a 10 μL solution was injected into the LC–MS system for analysis.

### Instrumentation

The chromatographic separation was performed using an Accela^TM^ U-HPLC system (Thermo Fisher Scientific, San Jose, CA, USA) comprising of a UHPLC pump and a PDA detector and the samples were scanned from 200 to 400 nm. The MS analysis was performed using a the LTQ Orbitrap XL hybrid instrument (Thermo Fisher Scientific, San Jose, CA, USA).

### Chromatographic conditions

The LC conditions were optimized as follows: column: Agilent SB-C_18_, 100 × 4.6 mm, 1.8 μm particle size (Agilent, USA); mobile phase: water with 0.1% formic acid (A), and methanol (B); flow rate: 300 μL/min; and injection volume: 10 μL; The gradient schedule was as follows: 10–13% B for 0–3 min, 13–65% B for 3–45 min, 65–70% B for 45–50 min. The ESI was operated in negative ion mode with a mass range of 125–800. For the non-target tandem Fourier transformation (FT-FT) mode, a data-dependent MS/MS (30K-15K) events scanning was used consisting of two scan events, a full-scan and MS/MS acquisitions. For the target FT-FT and IT-IT modes, the aimed parent ions were listed, and the collision energy was set in FT and IT mode, respectively. The set MS/MS isolation width was m/z 1.0 Da, maximum ion injection time was 50 ms, and the collision energy for the collision-induced dissociation (CID) was 30% of the maximum. Nitrogen and helium were used as the sheath and collision gasses, respectively. The key optimized MS parameters were as follows: source voltage: 3.5 kV; sheath gas (nitrogen): 45 L/min; auxiliary gas flow: 5 L/min; capillary voltage: −35.0 V; capillary temperature: 325.0°C; and tube lens: −110.0 V. The MS scan functions and the UHPLC solvent gradients were controlled using the Xcalibur data system (Thermo Fisher, San Jose, CA, USA). The data was collected and analyzed using the Xcalibur 2.07.

### Strategy for systematic analysis of absorbed constituents and metabolites

**Fig 1:**
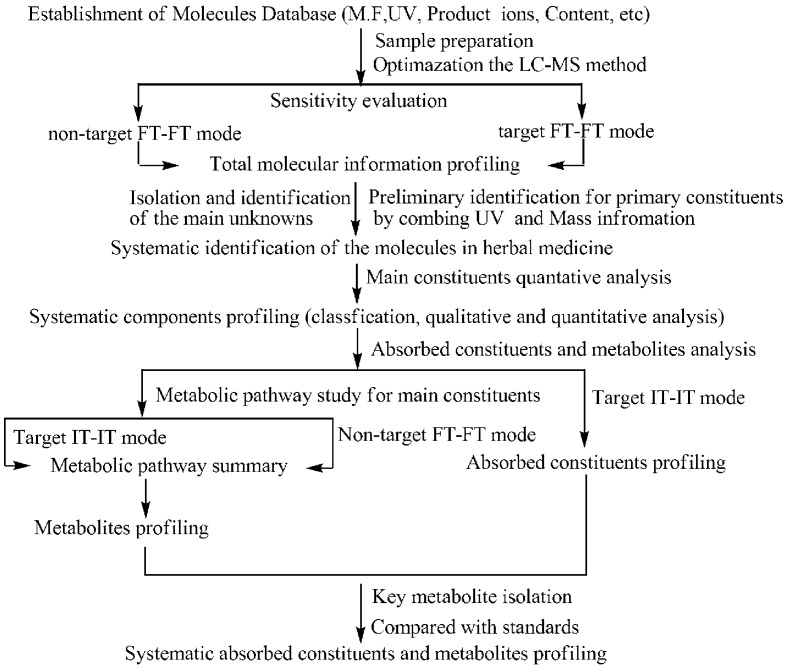
The strategy for systematic screening and identification of the absorbed constituents and metabolites in *S*. *glabra* by UHPLC–LTQ-Orbitrap

The strategy established for the systematic screening and identification of the absorbed components and their metabolites (Fig 1) involved three steps. First, previously known and published information on the components from previously studied herbal medicines including their molecular formulas and weight, characteristic product ions, UV absorption, and content were collected using the CAS SciFinder database. Then, we developed the LC-MS analytical method for the systematic identification of the components to facilitate the isolation and identification of the main unknown constituents, as well as the LC-MS/MS method for the simultaneous quantitative analysis to verify the main ingredients. Finally, we performed metabolism pathways studies with the representative constituents, as well as the key metabolites isolated, and then systematically profiled the absorbed constituents and their metabolites using different detection modes.

### Measurement of NO

The NO production was determined by measuring the amount of nitrite generated. Briefly, RAW264.7 macrophages were plated at a density of 5×10^4^ cells/mL in 96-well plates for 24 h, followed by treatment with LPS (1 μg/mL) and the components at different concentrations for an additional 24 h. The nitrite concentration was determined using an enzyme-linked immunosorbent assay (ELISA) kit according to the manufacturer's instructions. The relative cell viability of each parallel experimental group (n = 3) was expressed as a percentage (%) of the non-drug treated control, while the untreated controls were considered as 100% viable.

### Total RNA extraction and Reverse transcription- polymerase chain reaction (RT-PCR)

The RAW264.7 macrophages were cultured at a density of 2×10^5^ cells/mL in six-well plates overnigh, then incubated for 2h with a combination of different concentrations of the tested compounds, and then, the cells were further cultured for 12 h on treatment with LPS (1μg/mL). Then, the cells were rinsed with cold phosphate-buffered saline (PBS), and the total cellular RNA of the RAW264.7 cells was extracted using a Trizol Reagent kit according to instructions of the manufacturer. Total RNA (1 μg) was transcribed to cDNA using a Thermo Revert Aid First Strand cDNA Synthesis kit. The gene expressions of TNF-α, IL-1β, and IL-6 were amplified from the synthesized cDNA. The reverse transcription polymerase chain reaction (RT-PCR) was performed using the Roche FastStart Universal SYBR green master mix. The following primers were used for the PCR amplification: TNF-α, GTGTCCCAACA TTCATATT GTCAGT (forward) and TGGGAAGAGAAACCAGGGAGA (reverse); IL-6, GTCTTGGCCGAGGACTAAGG (forward) and TACTCGGCAAACCTAG TGCG (reverse); and IL-1β, TGGGATAGGGCCTCTCTTGC (forward) and CCATG GAATCCGTGTCTTCCT (reverse). Glyceraldehyde 3-phosphate dehydrogenase (GADPH) mRNA levels were used as the internal controls. The PCR reactions were carried out on the following schedule: 95°C for 10 min; 40 cycles at 95°C for 15 s, and the final extension was performed at 60°C for 1 min.

### Statistical Analysis

Data are expressed as means ± standard deviation (SD). A one-way analysis of variance (ANOVA) followed by Dunnett's t-test was applied to assess the statistical significance of the differences among the study groups (IBM^®^SPSS^®^ Statistics version 20, Chicago, USA). Avalue of P < 0.05 was chosen as the criterion of statistical significance.

## Results

### Systematic profiling of *S*. *glabra* water extract constituents

We have developed a rapid method based on a UHPLC-MS/MS analysis for the comprehensive identification of the constituents of *S*. *glabra* [3]. However, the sensitivity of the established method was limited and, therefore, not adequate for further *in vivo* analysis. Therefore, we optimized an analytical method that was suitable for the simultaneous analysis of the absorbed herbal components and metabolites, and operated it in in two different detection modes. In the first mode, both the accurate MS and MS/MS scan events were performed using a data dependent scan mode with the Orbitrap detector. This mode is described as a non-target FT-FT mode. In the alternative mode referred as the target FT-FT mode, the data dependent scanning was operated by using a parent ion list to detect the specified or target constituents. The superiority of the previous scan mode enabled the acquisition of both the accurate parent and product ions information simultaneously for main unexpected components identification. In contrast, the latter mode was more sensitive for the detection of the specified components reported by previous studies on the herb that were not detected using the first mode due to interference or its lower sensitivity. The established method was also sensitive enough for metabolite analysis, as illustrated in S1 Table and most of the representative standards showed a signal to noise ratio (S/N) that was higher more than 1000 even at a concentration of 500 ng/mL. As a result, 89 molecules were profiled from *S*. *glabra* (S2 Table), and 42 of them were definitely identified by comparing their retention times with those of the standards, including all the main peaks (see S2 File). Taken together, these results and those of the previous quantitative analysis [4] showed that the main components of *S*. *glabra* could be profiled into four categories: caffeoylquinic acids (3-*O*-caffeoylquinic acid, 5-*O*-caffeoylquinic acid and 4-*O*-caffeoylquinic acid), coumarins (isofraxidin and eleutheroside B_1_), flavonoids (neoastilbin, astilbin, neoisoastilbin, isoastilbin and quercetin-3-*O*-*β-*D-glucuronide), and dicaffeoyl derivatives (rosmarinic acid-4-*O*-*β-*D-glucoside and rosmarinic acid).

### Characterization of the absorbed constituents of *S*. *glabra* in rat plasma

To detect the absorbed constituents of *S*. *glabra*, we compared the retention times and mass information obtained from the biological sample with those obtained from the extract. The sensitivity of the analytical method needs to be higher than that of conventional methods because of the poor absorption characteristics nature of most natural products. Since the retention time, as well as parent and characteristic product ions information were obtained for the crude drug, we used a parent ion-dependent list strategy for the identification using the more sensitive IT mass detector (referred to as target IT-IT mode). In addition, the collision energy of each parent ion was simultaneously set to obtain the characteristic product ions information. The target IT-IT mode performed a selective scan and minimized the interference from the molecular ions of the complex matrix components. As shown in S1 Table, most of the representative constituents were detected even at a concentration of 0.5 ng/ml using target IT-IT detection mode. By comparing the retention time of the extract ion peak compared with that obtained from the crude drug, the absorbed constituent was confidently identified. As a result, 54 components were absorbed into the plasma, and 36 of them were positively identified by comparing their retention times and mass information with those of the obtained standards (see S2 File and S2 Table).

### Characterization of phase I and phase II metabolites in rat plasma

**Fig 2:**
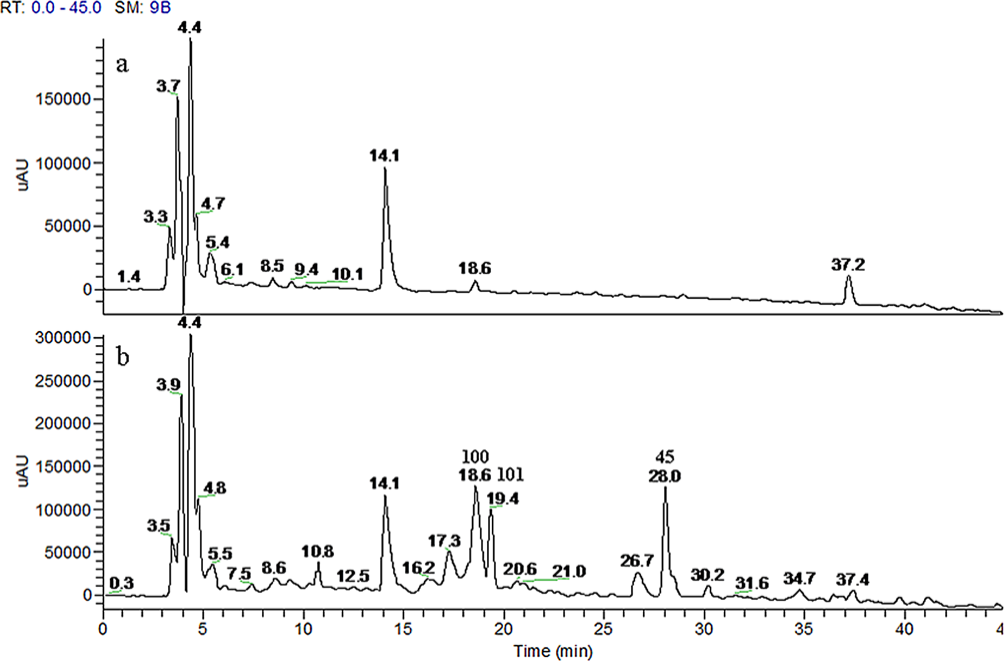
UHPLC-DAD chromatograms: (a) blank plasma; (b) drug-containing plasma after oral administration of *S*. *glabra*.

**Fig 3:**
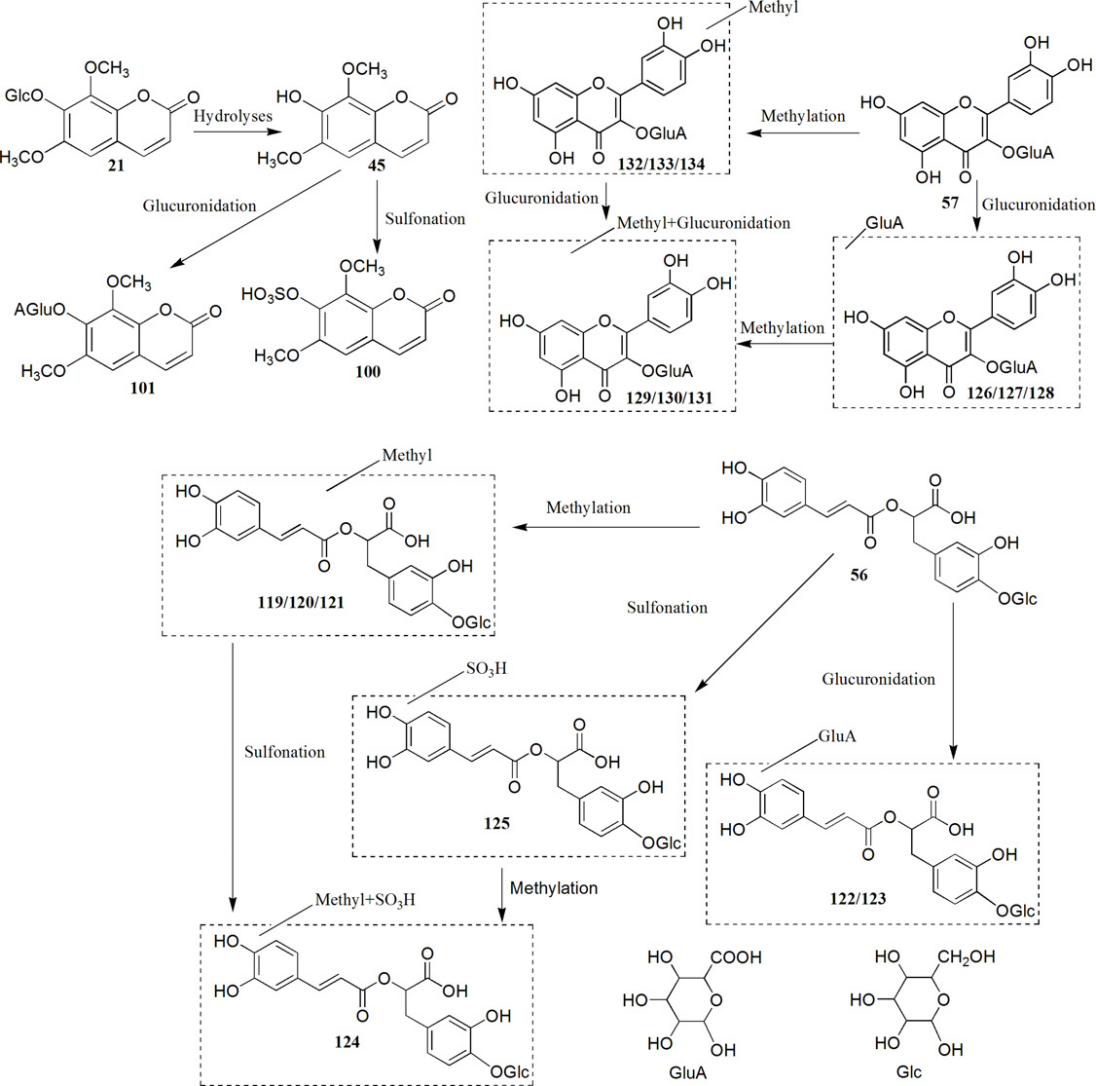
The proposed metabolic pathways of eleutheroside B_1_ (21), rosmarinic acid-4- O-*β*-D-glucoside (56) and quercetin-3-*O*-*β-*D-glucuronide (57) in rat plasma after oral administration.

To systematically identify the phase I and phase II metabolites of the absorbed components and determine their parent compound origins in the herbal medicine, we firstly used a non-target FT-FT detection mode detect the main metabolites in the drug-containing plasma.As is shown in Fig 2, only three main peaks (**45**, **100** and **101**) were obviously detected when compared with blank plasma sample. To acquire additional absorption information, we further studied the metabolic pathways of seven of the representative main constituents (**18**, **21**, **45**, **54**, **56**, **57** and **64**) in *S*. *glabra* using a target IT-IT mode. Furthermore, the parent ion of the possible metabolites were listed and the collision energy was set to obtain the corresponding predicted product ions information (S3 Table). The results showed that all the representative constituents except eleutheroside B_1_ (**21**) were obviously detected in the rat plasma. In addition, 43 metabolites were identified, except for the known metabolites as previously reported in the literatures [9–15] and 18 metabolites (**100**, **101** and **119**–**134)** were reported for the first time (Table 1). The coumarin aglycones such as isofraxidin (**45**), were firstly absorbed into plasma, and then mainly metabolized by the glucuronidation and sulfation pathways, while the coumarin-O-glycosides such as eleutheroside B_1_ (**21**), were first degraded and absorbed as their aglycone forms, then metabolized into glucuronidation and sulfation conjugates. The dicaffeoyl aglycone and its glycosylation conjugates including rosmarinic acid (**64**) and rosmarinic acid-4-O-β-D- glucoside (**56**), were both absorbed in their unchanged, methylated, glucuronidated, and sulfated forms *in vivo*, but rosmarinic acid (**64**) was not detected after a single oral administration of **56**. This observation indicates that the absorption mechanisms likely differ between the dicaffeoyl derivatives and coumarins. The special flavonoid glycoside such as astilbin (**54**) and quercetin-3-*O*-*β-*D-glucuronide (**57**), they were not detected as their aglycone forms in plasma, while phase I and II reactions including methylation, glucuronidation and sulfation were the main metabolic pathways. This result indicates that the conjugation of the flavonoid nucleus with a rhamnose or glucuronide may confer different biological activities compared with those of the aglycone since they have different metabolic pathways *in vivo*. The metabolic pathways of **21**, **56,** and **57** are summarized in Fig 3. The identified metabolite mass information of the seven representative constituents, as well as that of the other main constituents of *S*. *glabra* were listed and scanned for in the rat plasma after oral administration of *S*. *glabra* extract using a targeted IT-IT dectection mode. The results revealed that 23 absorbed metabolites (**18**, **26**, **45**, **56**, **64**, **91**, **92**, **96**–**101**,**103**, **105**, **106**, **108**, **109**, **120**, **121**, **124**, **128,** and **134)** were profiled and determined to have originated from the six main representative constituents (Table 1). In addition, the three main peaks detected in the rat plasma (**45**, **100** and **101**) were deduced to have originated from eleutheroside B_1_ and isofraxidin. Since **100** and **101** were detected in rat urine following the oral administration of **45**, we collected the drug-containing urine, and then isolated and purified both metabolites. Subsequently, **100** and **101** were further identified to be isofraxidin -7-O-sulfate and isofraxidin-7-O-α-D-glucuronide, respectively. The description of the isolation process, as well as the proton (^1^H)-NMR and high-resolution (HR)-ESI-MS data of **100** and **101** are presented as S1 File. The phase II metabolites of astilbin (**54**) were not detected in the rat plasma following oral administration of the water extract, likely because astilbin is not stable [16] and has a low content in boiling water extract.

**Table 1:** Detected metabolites of 18, 21, 45, 54, 56, 57 and 64 in rat plasma after oral administration in single or extract forms.

t_*R*_ (min)	Parent ion	MS/MS data	Identification	Origination	^a^ Plasma 1	^b^ Plasma 2
18.0	353.1	191.1(100), 178.8(25),173.0(36)	**5-*O*-caffeoylquinic acid** (**18**) ^c^	**18**	√	√
21.1	179.1	135.0 (100)	caffeic acid (**26**) ^c^	**18**, **64**	√	√
17.9	355.1	311.1 (100), 179.0 (38)	caffeic acid glucuronide 1 (**91**)	**18**	√	√
18.9	355.1	311.2 (100), 179.1 (30), 175.0 (15)	caffeic acid glucuronide 2 (**92**)	**18**	√	√
17.3	259.1	179.1 (100), 128.0 (38)	caffeic acid sulfate 1 (**93**)	**18**, **64**	√	√
18.2	273.1	193.1(100), 229.0 (16)	methylated caffeic acid sulfate 1 (**96**)	**18**, **64**	√	√
18.9	273.1	193.1(100), 229.0 (17)	methylated caffeic acid sulfate 2 (**97**)	**18**, **64**	√	√
27.0	163.0	119.0 (100),	*p*-coumaric acid (**98**) ^c^	**18**	√	√
27.5	165.0	147.0 (100), 121.1 (32)	3-Hydroxyphenylpropionic acid (**99**)	**18**	√	√
			**eleutheroside B**_**1**_ (**21**)	**21**	×	√
28.2	221.0	206.0 (100)	isofraxidin **(45)**	**21**, **45**	√	√
19.1	301.0	221.1 (100)	isofraxidin-7-O-sulfate (**100**) ^c^^,^ ^d^	**21**, **45**	√	√
19.5	397.1	221.1 (100)	isofraxidin -7-O-α-D-glucuronide (**101**) ^c^^,^ ^d^	**21**, **45**	√	√
28.1	221.0	206.0 (100)	**isofraxidin** (**45**) ^c^	**21**, **45**	√	√
18.7	301.0	221.1 (100)	isofraxidin-7-O-sulfate (**100**) ^c^^,^ ^d^	**21**, **45**	√	√
19.1	191.1	162.9 (100), 135.0 (8)	isofraxidin deoxymethyl conjugation	**45**	√	×
19.6	397.1	221.1 (100), 175.0 (10)	isofraxidin -7-O-α-D-glucuronide (**101**) ^c^^,^ ^d^	**21**, **45**	√	√
34.0	359.1	161.0 (100), 178.9(32), 197.2(20)	**rosmarinic acid** (**64**) ^c^	**64**	√	√
28.0	535.1	359.1 (100)	rosmarinic acid glucuronide 1 (**102**)	**64**	√	×
31.1	535.1	359.1 (100)	rosmarinic acid glucuronide **2** (**103**)	**64**	√	√
31.1	521.1	359.0 (100)	rosmarinic acid-4-*O*-*β-*D-glucoside (**56**) ^c^	**56, 64**	√	√
29.0	439.1	359.1 (100), 259.2 (80)	rosmarinic acid sulfate (**104**) ^c^	**64**	√	×
32.3	549.1	373.2 (100), 161.1 (12)	methylated rosmarinic acid glucuronide 1 (**105**)	**64**	√	√
36.0	549.1	373.2 (100), 337.1(60)	methylated rosmarinic acid glucuronide 2 (**106**)	**64**	√	√
36.2	373.1	179.2 (64), 160.9(100), 135.0 (60)	methylated rosmarinic acid (**107**)	**64**	√	×
18.1	259.1	179.0 (100), 214.9 (11)	caffeic acid sulfate 2 (**108**)	**64**	√	√
12.5	261.1	181.1(100), 217.1 (86)	dihydrocaffeic acid sulfate (**109**)	**64**	√	√
30.2	449.1	303.2 (100), 285.2(46)	**astilbin** (**54**) ^c^	**54**	√	√
15.8	625.1	479.1 (46), 449.2 (78), 303.1 (100), 285.0 (39)	astilbin glucuronide 1 (**110**)	**54**	√	×
16.8	625.1	479.1 (52), 449.2 (49), 303.1 (100), 285.0 (28)	astilbin glucuronide 2 (**111**)	**54**	√	×
21.7	625.1	479.1 (34), 449.2 (72), 303.1 (100), 285.0 (28)	astilbin glucuronide 3 (**112**)	**54**	√	×
23.5	625.1	479.1 (59), 449.2 (100), 303.1 (60), 285.0 (41)	astilbin glucuronide 4 (**113**)	**54**	√	×
35.0	463.1	299.1(100), 317.2 (389)	3'-O-methylated astilbin (**114**)	**54**	√	×
19.0	639.1	493.0 (21), 463.2 (100), 317.2(33),299.2 (12)	methylated astilbin glucuronide 1 (**115**)	**54**	√	×
20.0	639.1	493.0 (12), 463.2 (100), 317.2(35),299.2 (11)	methylated astilbin glucuronide 2 (**116**)	**54**	√	×
24.7	639.1	493.0 (14), 463.2 (100), 317.2(43),299.0 (11)	methylated astilbin glucuronide 3 (**117**)	**54**	√	×
26.2	639.1	493.0 (15), 463.2 (100), 317.2(15),299.1 (10)	methylated astilbin glucuronide 4 (**118**)	**54**	√	×
31.5	521.0	359.1 (100)	**Rosmarinic acid-4-*O*-*β-*D-glucoside** (**56**)	**56**	√	√
33.7	535.1	373.1(46), 359.2(100), 341.1(43)	methylated rosmarinic acid-4-O-β-D-glucoside 1 (**119**) ^d^	**56**	√	×
35.1	535.1	373.1(27), 359.2(100), 341.1(29)	methylated rosmarinic acid-4-O-β-D- glucoside 2 (**120**) ^d^	**56**	√	√
35.7	535.1	373.1(100)	methylated rosmarinic acid-4-O-β-D- glucoside 3 (**121**) ^d^	**56**	√	√
26.8	697.1	521.0 (100), 359.2 (22)	rosmarinic acid-4-O-β-D-glucoside glucuronide 1 (**122**) ^d^	**56**	√	×
28.6	697.1	521.0 (100), 359.2 (62)	rosmarinic acid-4-O-β-D-glucoside glucuronide 1 (**123**) ^d^	**56**	√	×
30.6	615.1	535.2 (35), 453.1 (17), 393.2 (38), 373.2 (27), 273.1 (100)	methylated rosmarinic acid-4-O-β-D-glucoside sulfate (**124**) ^d^	**56**	√	√
29.3	601.1	521.1(100), 359.1(53), 259.0 (22)	rosmarinic acid-4-O-β-D-glucoside sulfate (**125)** ^d^	**56**	√	×
31.9	477.1	301.0 (100)	**quercetin-3-*O*-*β-*D-glucuronide** (**57**) ^c^	**57**	√	√
23.2	653.2	477.1 (100), 301.1 (42)	quercetin-3-*O*-*β-*D-glucuronide glucuronide 1 (**126)** ^d^	**57**	√	×
26.3,	653.2	477.2 (100), 301.1 (18)	quercetin-3-*O*-*β-*D-glucuronide glucuronide 2 (**127)** ^d^	**57**	√	×
29.4	653.2	477.2 (100), 301.1 (14)	quercetin-3-*O*-*β-*D-glucuronide glucuronide 3 (**128)** ^d^	**57**	√	√
23.9	667.2	491.1(100), 315.1(19)	methyl quercetin-3-*O*-*β-*D-glucuronide glucuronide 1 (**129)** ^d^	**57**	√	×
26.0	667.2	491.1 (100), 315.0 (30)	methyl quercetin-3-*O*-*β-*D-glucuronide glucuronide 2 (**130)** ^d^	**57**	√	×
31.9	667.2	491.2 (100)	methyl quercetin-3-*O*-*β-*D-glucuronide glucuronide 2 (**131)** ^d^	**57**	√	×
34.6	491.1	315.1 (100)	methyl quercetin-3-*O*-*β-*D-glucuronide 1 (**132)** ^d^	**57**	√	×
35.2	491.1	315.2 (100)	methyl quercetin-3-*O*-*β-*D-glucuronide2 (**133)** ^d^	**57**	√	×
36.6	491.1	315.2 (100)	methyl quercetin-3-*O*-*β-*D-glucuronide 3 (**134)** ^d^	**57**	√	√
30.3	193.0	178.0 (81), 161.0 (100), 148.8 (72),134.0 (52)	isoferulic acid (**135**) ^c^	**18**	√	×
28.6	193.0	178.1 (58), 161.0 (34), 149.1(100),134.0 (63)	ferulic acid (**136**) ^c^	**64**	√	×

^a^Refer to the collected plasma after oral administration of the respective standard

^b^Refer to the collected plasma after oral administration of the extract of *S*. *glabra*

^c^Metabolites were identified by comparing the retention time of reference standards

^d^ Metabolites reported firstly

### Anti-inflammatory activities evaluation

**Fig 4:**
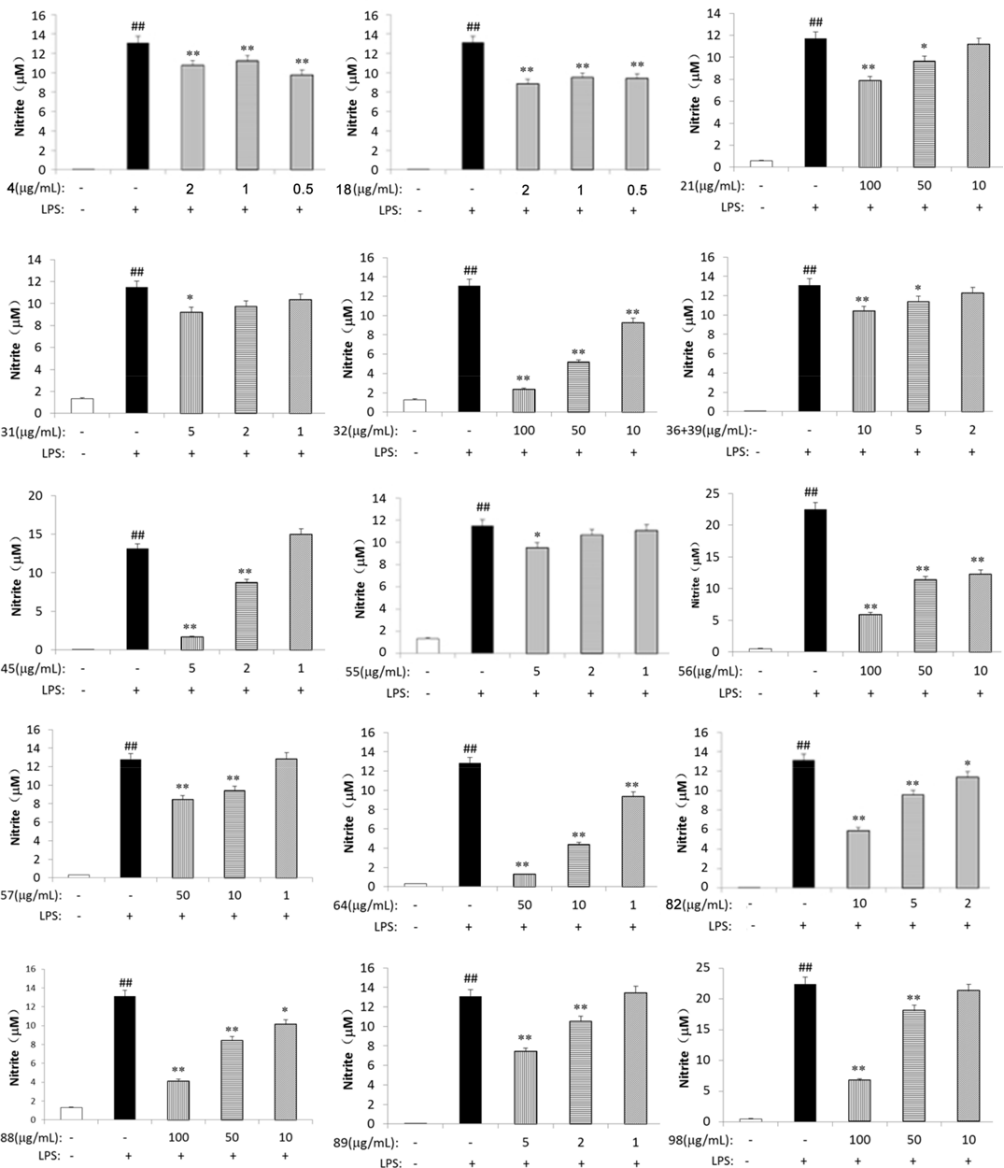
Effects of 15 metabolites (4, 18, 21, 31, 32, 36+39, 45, 55–57, 64, 82, 88, 89 and 98) on NO production in LPS-stimulated RAW264.7 macrophages. Cells were treated with LPS (1μg/ml) in the absence or presence of the constituents at different concentrations for 24 h. Amounts of NO were determined using the Griess reagent kits; ++p<0.01,control group vs LPS-stimulated group; +p<0.05,control group vs LPS-stimulated group;**P<0.01, LPS vs LPS plus constituents-treated group. B. Values shown in the graphs are mean ± standard deviation (n = 3).

**Fig 5:**
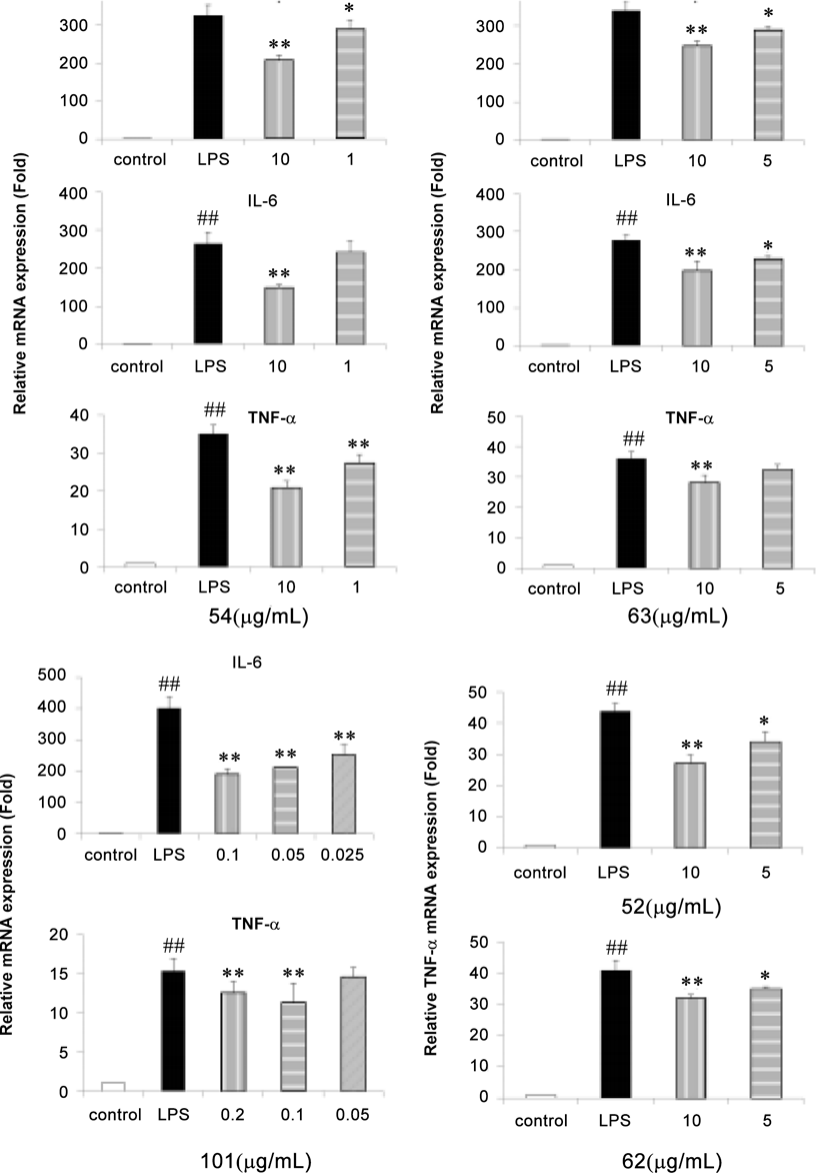
Effects of 52, 54, 62, 63 and 101 on inflammation-related gene expression in LPS-stimulated RAW 264.7 macrophages. Cells were treated with LPS (1μg/mL) in the absence or presence of the constituents at different concentrations for 10 h. The mRNA level was analyzed using real-time PCR; ++p<0.01, control group vs LPS-stimulated group; +p<0.05, control group vs LPS-stimulated group; **P<0.01, LPS vs LPS plus constituents-treated group. Values shown in the graphs are mean ± standard deviation (n = 3).

Ten absorbed metabolites (**11**, **18**, **26**, **32**, **44**, **45**, **54**, **57**, **64**, and **98**) of *S*. *glabra* were reported to possess anti-inflammatory activities [8, 17–25]. Twenty-four metabolites (**1**, **4**, **18**, **21**, **22**, **26**, **31**, **32**, **36**+**39**, **45**, **52**, **54**, **55**–**57**, **62**, **63**, **73**, **82**, **86**, **88**, **89**, **98** and **101**) were evaluated for their anti-inflammatory potential by measuring NO production in LPS-stimulated RAW264.7 macrophages. The cell viability was firstly evaluated using the MTT assay as previous described [8]. Fifteen of the tested compounds (**4**, **18**, **21**, **31**, **32**, **36+39**, **45**, **55–57**, **64**, **82**, **88**, **89**, and **98)** inhibited the LPS-induced NO production at concentration of 0.5–100 μg/mL (Fig 4). The effects of five components (**52**, **54**, **62**, **63** and **101)**, which lacked NO inhibitory activities, on the mRNA expression of the pro- inflammatory cytokines IL-1β, IL-6 and TNF-α mRNA expression were evaluated using RT-PCR. As shown in Fig 5, **54** obviously inhibited IL-1β, IL-6 and TNF-α mRNA expression, which was consistent with a previous report [25], while its isomer **63** showed similar but weaker activities. In contrast, the other configuration isomers of **54** (**52** and **62**) only inhibited TNF-α mRNA expression in our study. Isofraxidin-7-O-α-D-glucuronide (**101**), the phase II metabolite of isofraxidin (**45**), did not show inhibitory effect of NO production, but inhibited both IL-6 and TNF-α mRNA expression at the concentration of 0.025–0.2 μg/mL.

## Discussion

Although most TCM formulations and prescriptions have a multi-constituent characteristic, those used for therapeutic purposes should be limited because of their poor absorption, low bioavailability, and low content of the raw herbs. In addition, the decoction, which is the traditional method of preparation of TCM formulations, excludes numerous poorly water-soluble components [26]. Therefore, the well- absorbed and bioactive metabolites groups should contributed to their main therapeutic effects. Whether the absorbed constituents and their metabolites are bioactive is also difficult to verify since these compounds have multiple targets, and it would be unrealistic to attempt to obtain and test every metabolite individually. In this study, we used a single inflammatory cell model with four evaluation markers, which were NO, IL-1β, IL-6, and TNF-α. Most of the studied constituents, including the both absorbed components and their phase I or II metabolites, were determined to be bioactive, indicating that the anti-inflammatory constituents in this herbal medicine are very extensive. Moreover, the anti-inflammatory effect of herbal medicines may play an important role in preventing and treating chronic inflammatory diseases by targeting multiple pathways.

## Conclusion

In this study, we established an analytical strategy for the first time based on a UHPLC system coupled with LTQ-Orbitrap mass spectrometry methods using different detection modes for the systematic analysis and profiling of the main bioactive components of *S*. *glabra* and their potential metabolites in rat plasma. We demonstrated that 54 components of *S*. *glabra* were ultimately absorbed into the plasma following oral administration, and 36 of them were positively identified. Moreover, 23 metabolites were characterized after oral administration of *S*. *glabra* extract, and their origins were traced. The metabolism of the phenolic glycosides (**21**, **56**, and **57**) was first studied, and phase II reactions (including glucuronidation and sulfation) were their main metabolic pathways. Twenty of 24 screened components exhibited anti-inflammatory activities, while the effects of 15 components (**4**, **21**, **31**, **36+39**, **52**, **55**, **56**, **57**, **62, 63**, **82**, **88**, **89**, **98**, and **101)** against NO production or IL-1β, IL-6 and TNF-α mRNA expression were first reported here. Based on these results a variety of absorbed constituentsof *S*. *glabra*, including organic acids (**4**), caffeoyl derivatives (**18**, **32**, **56**, **64**, and **82**), coumarins (**21**, **31**, and **45**), flavonoids (**36**+**39**, **57**, **52**, **54**, **62**, **63**, and **88**), sesquiterpenoids (**56** and **89**) and their main metabolites (**98** and **101**) exert anti- inflammatory activities. Furthermore, the established novel strategy is a potentially powerful tool for systematically screening and characterizing of the absorbed constituents and metabolites of TCM formulations and, therefore, provides an efficient shortcut for screening natural-based medicines and products for potential anti-inflammatory agents.

## Supporting Information

S1 File NMR data of compounds 44, 57, 88, 100, 101 and MS data of 100 and 101. (PDF)

S2 File UHPLC Chromatograms of the standards identified in *S*. *glabra*. (PDF)

S1 Table Sensitivity evaluation of representative constituents from *S*. *glabra*. (PDF)

S2 Table Profiling of constituents in *S*.*glabra* and the absorbed constituents in rat plasma by UHPLC-LTQ/Oribtrap. (PDF)

S3 Table Predicted metabolites of 18, 21, 45, 54, 56, 57 and 64 in rat plasma after oral administration. (PDF)
