# Supplementary material for: Systematic Analysis of Absorbed Anti-Inflammatory Constituents and Metabolites of Sarcandra glabra in Rat Plasma Using Ultra-High-Pressure Liquid Chromatography Coupled with Linear Trap Quadrupole Orbitrap Mass Spectrometry
Source: PLoS One. 2016 Mar 14;11(3):e0150063. doi: 10.1371/journal.pone.0150063 (PMC4790918; doi:10.1371/journal.pone.0150063)
Supplement: S1 File — (PDF) [file pone.0150063.s001.pdf]

NMR data of compounds 44, 57, 88, 100, 101 and MS data of 100 and 101

1. Compound **57** (quercetin-3-*O*-*β*-D-glucuronide)

UV λ_{max} (MeOH) nm : 256, 355 nm

^{1}H-NMR(400 MHz, DMSO-d_6 : 12.54 (1H, s, 5-OH) , 10.87 (1H, s,7-OH) , 9.74 (1H, s, 4'-OH) , 9.21(1H, s, 3'-OH) , 6.39(1H, brd, *J* = 2.0 Hz, H-8) , 6.19 (1H, brd, *J* = 2.0 Hz, H-6) , 7.51 (1H, brd, *J* = 2.0 Hz, H-2') , 6.82 (1H, d, *J* = 8.4 Hz, H-5') , 7.58 (1H, dd, *J* = 8.4 , 2.0 Hz, H-6') , 5.48 (1H, d, *J* = 7.2 Hz, H-1'').^{13}C-NMR (100 MHz, DMSO-d_6) : 156.3(C-2), 133.1 (C-3), 177.3(C-4) , 161.3(C-5) , 98.9(C-6) , 164.3 (C-7) , 93.7 (C-8) , 156.4 (C-9) , 101.2 (C-10) , 121. 8 (C-1') , 116.1(C-2') , 145.0 (C-3') , 148.7 (C-4') , 115.3 (C-5'), 120.9 (C-6'), 103.9 (C-1'') , 73.9 (C-2'') ,76.1(C-3''), 71.4 (C-4'') , 75.9(C-5''), 169.8(C-6'').

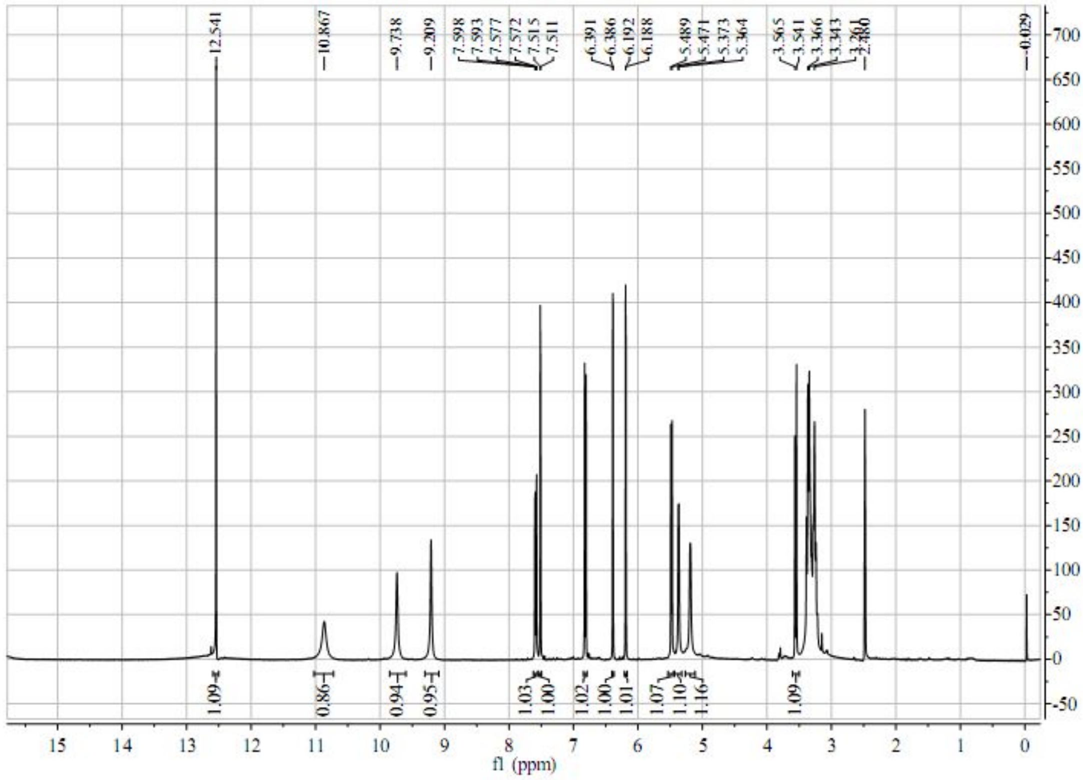

^{1}H-NMR(400 MHz, DMSO-d_6) of **57**

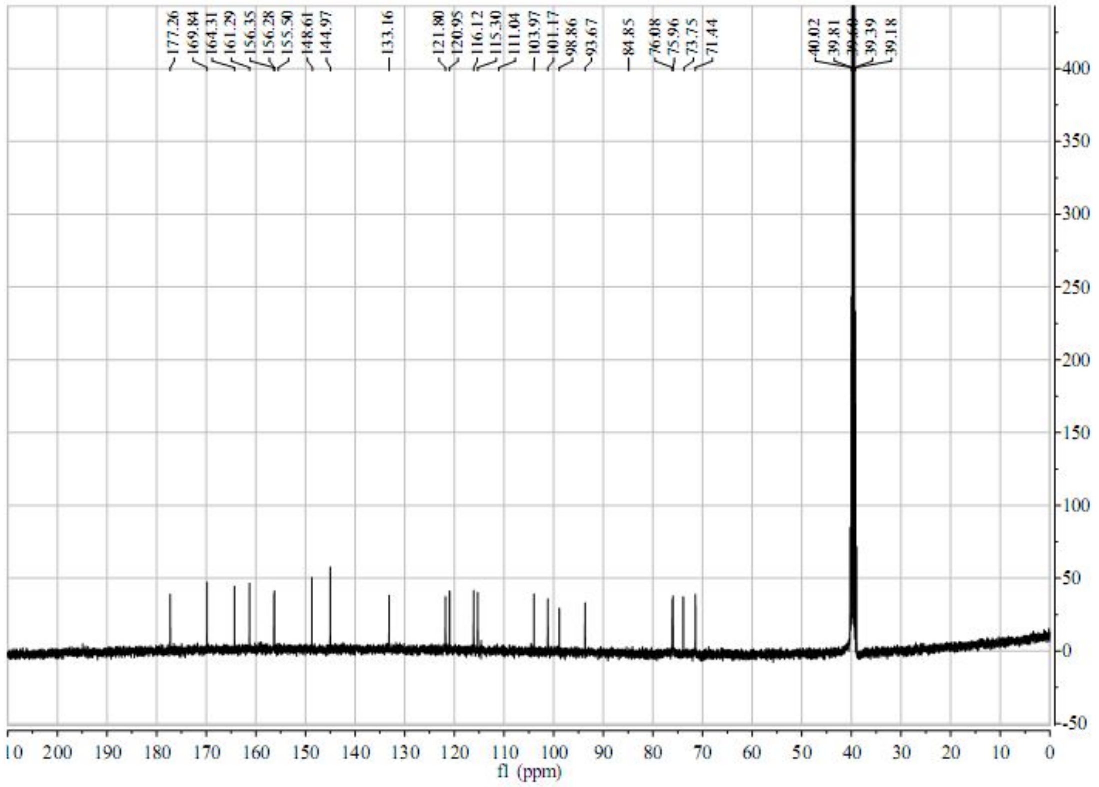

^{13}C-NMR of **57** (100 MHz, DMSO-*d*$_6$)

2. Compound **88** (5-hydroxy-7,8-dimethoxy-flavanone)

UV λ_{max} (MeOH) nm: 256, 355 nm

^{1}H-NMR (300 MHz, DMSO-*d*$_6$, δ) : 3.03(1H, dd, $J = 12.6$ and 16.8 Hz,3-Hα), 2.78(1H, dd, $J = 16.5$ and 3.0 Hz, 3-H$_\beta$), 5.50 (1H, dd, J = 12.6 Hz and 3.0 Hz, 2-H),3.77(3H, s, 7-OCH$_3$), 3.81 (3H, s, 8- OCH$_3$), 6.18(1H, s, H-6), 7.35-7.56(5H, m, H-2', 3', 4', 5', 6'). ^{13}C-NMR (75 MHz, DMSO-*d*$_6$, δ) : 189.9 (C-4), 78.7 (C-2), 44.5(C-3), 54.4 (C-8- OCH$_3$), 54.4 (C-8- OCH$_3$), 60.0 (C-7- OCH$_3$), 157.6(C-5), 157.5 (C-7), 156.3 (C-9), 92.4(C-6), 138.7 (C-8), 104.3(C-10), 129.0 (C-1'), 127.9 (C-2', 6'), 127.7 (C-4'), 125.4(C-3', 5').

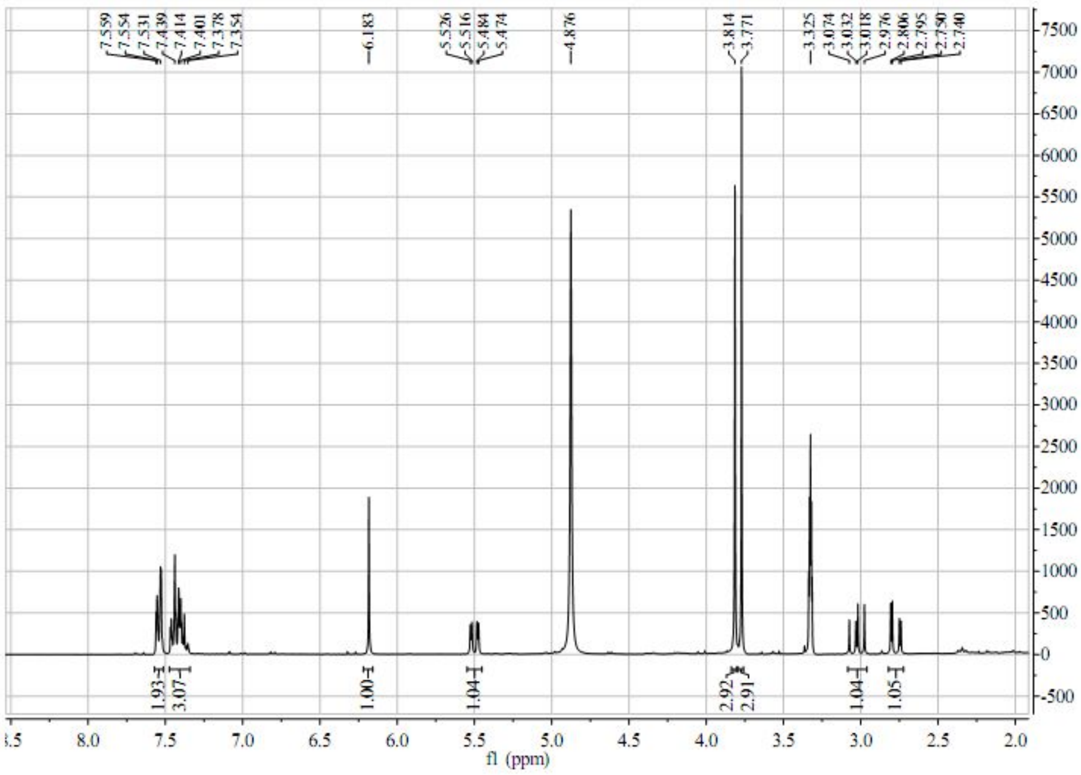

^{1}H-NMR (300 MHz, DMSO-d_6) of **88**

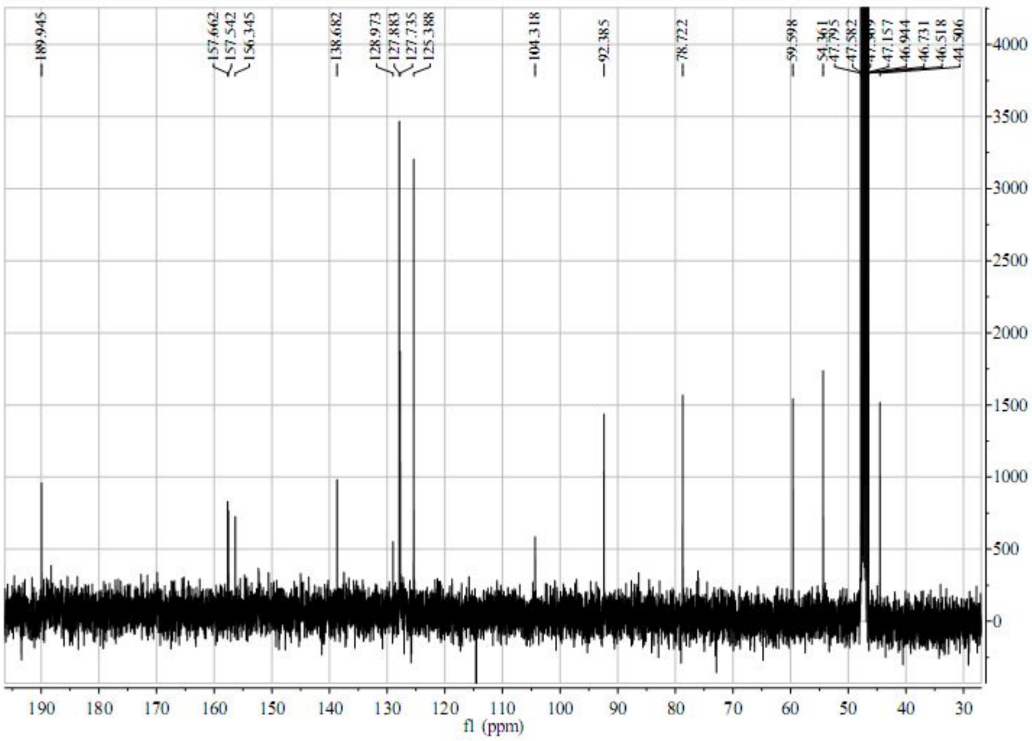

^{13}C-NMR (75 MHz, DMSO-d_6) of **88**

3. Compound **100** (isofraxidin-7-O-sulfate)

HR-ESI-MS m/z 301.00235 $[M-H]^-$;, MS^2: 221.08334. ^1H-NMR (400 MHz, DMSO-d_6) δ: 6.40 (1H, d, J = 8.8 Hz, H-3), 7.96 (1H, d, J = 10.0 Hz, H-4), 7.02 (1H, s, H-5), 3.95(3H,s, OCH_3-8), 3.76 (3H,s, OCH_3-6)。

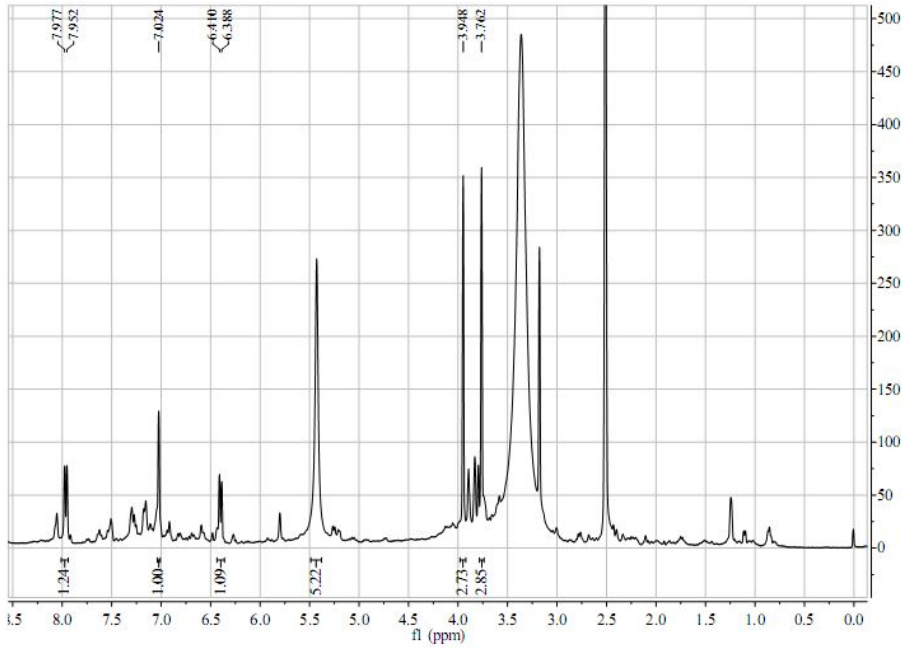

^1H-NMR (400 MHz, DMSO-d_6) of **100**

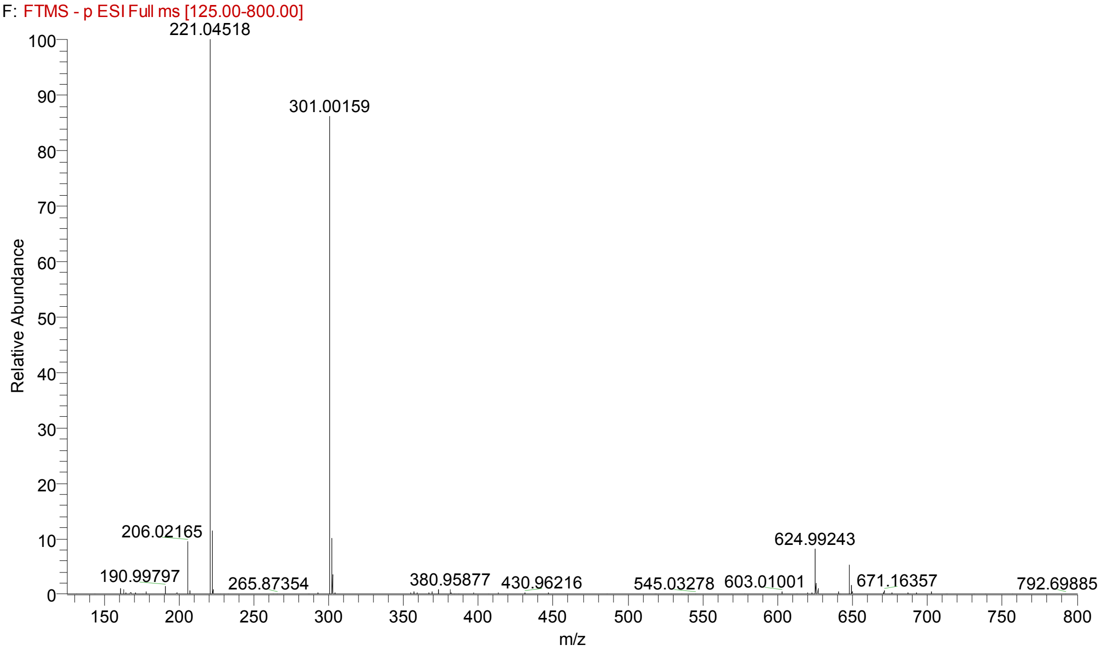

HR-ESI-MS of **100**

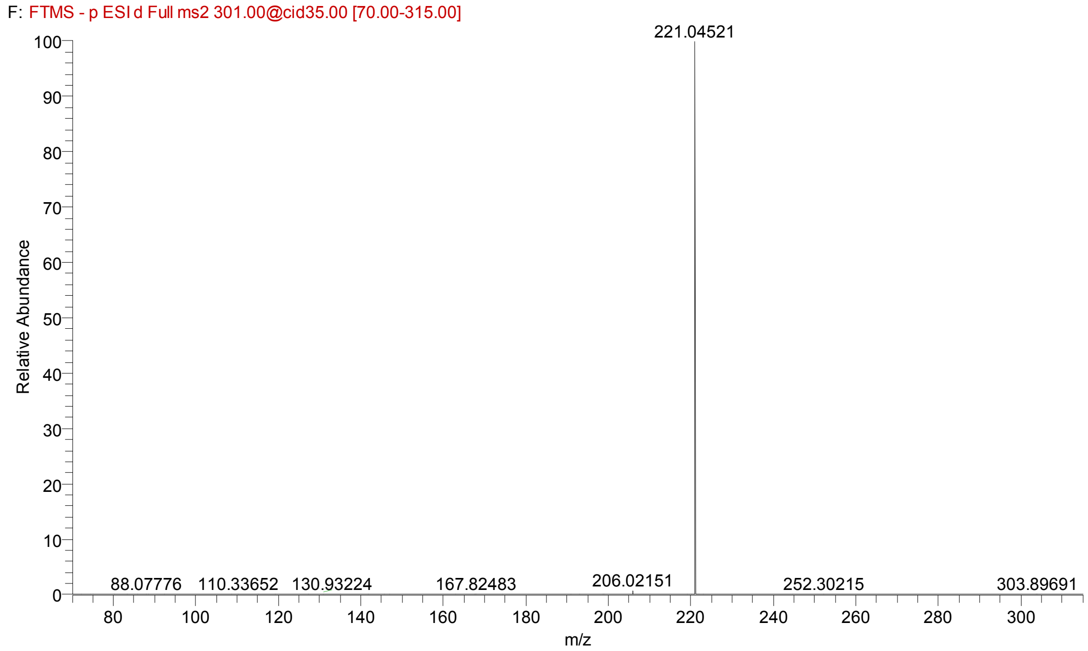

HR-ESI-MS/MS of **100** (Product ion of m/z 301)

4. Compound **101** (isofraxidin -7-O-α-D-glucuronide)

HR-ESI-MS m/z 397.07623 $[M-H]^-$; MS^2: 221.04536。^{1}H-NMR (400 MHz, DMSO-d_6) δ: 6.41 (1H, d, J = 9.6 Hz, H-3), 7.80 (1H, d, J = 9.6 Hz, H-4), 7.15(1H, s, H-5), 3.97(3H,s, OCH_3-8), 3.83(3H,s, OCH_3-6), 5.15 (1H, d, J = 4.8 Hz, H-1').

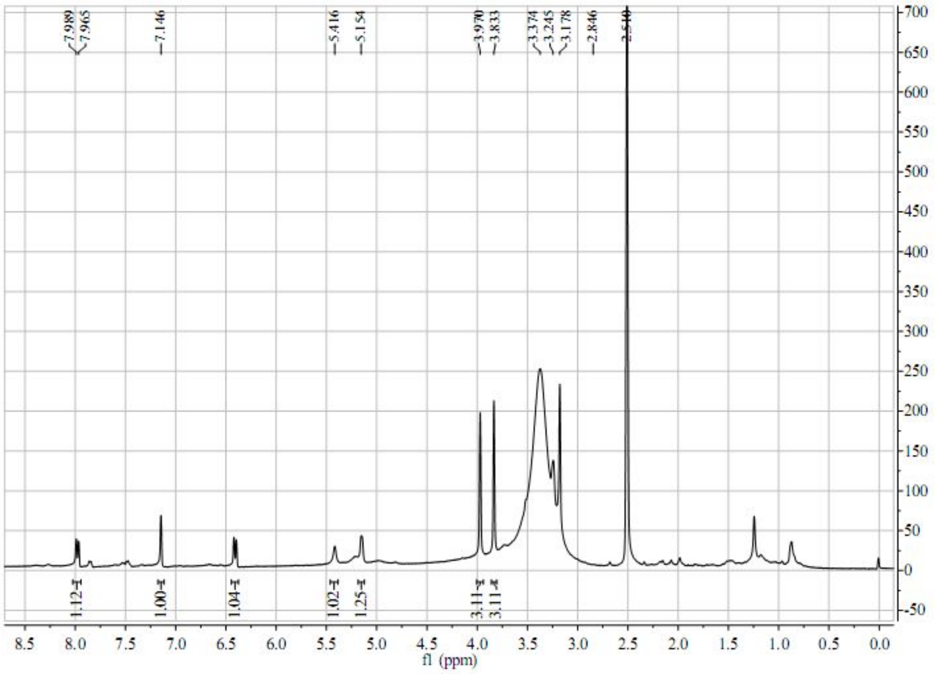

^{1}H-NMR (400 MHz, DMSO-d_6) of **101**

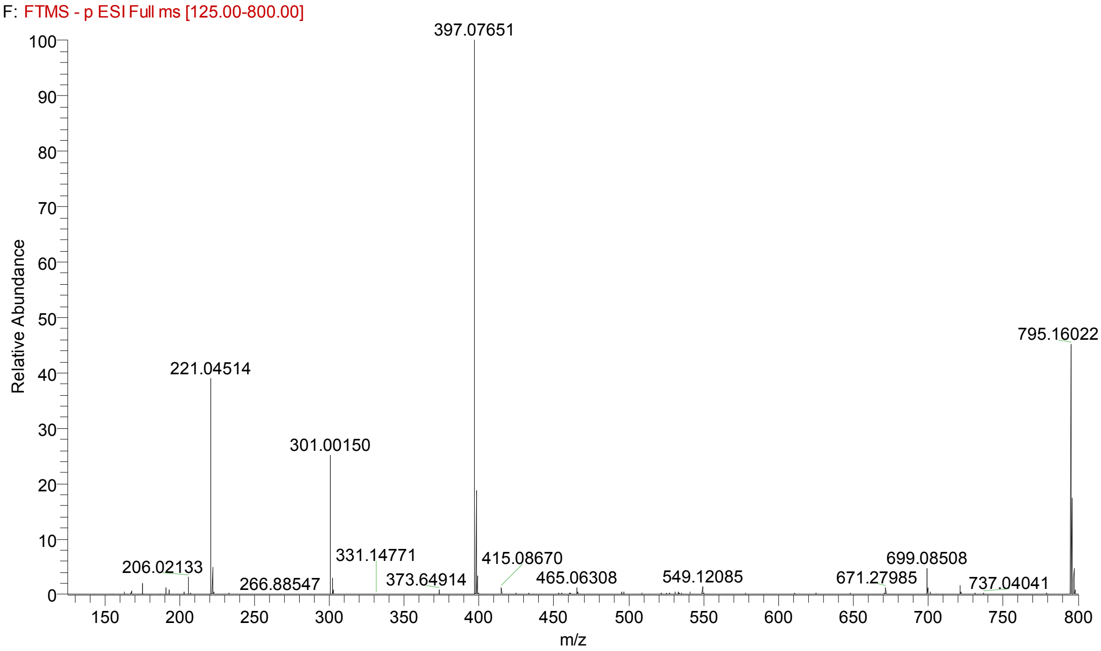

HR-ESI-MS of **101**

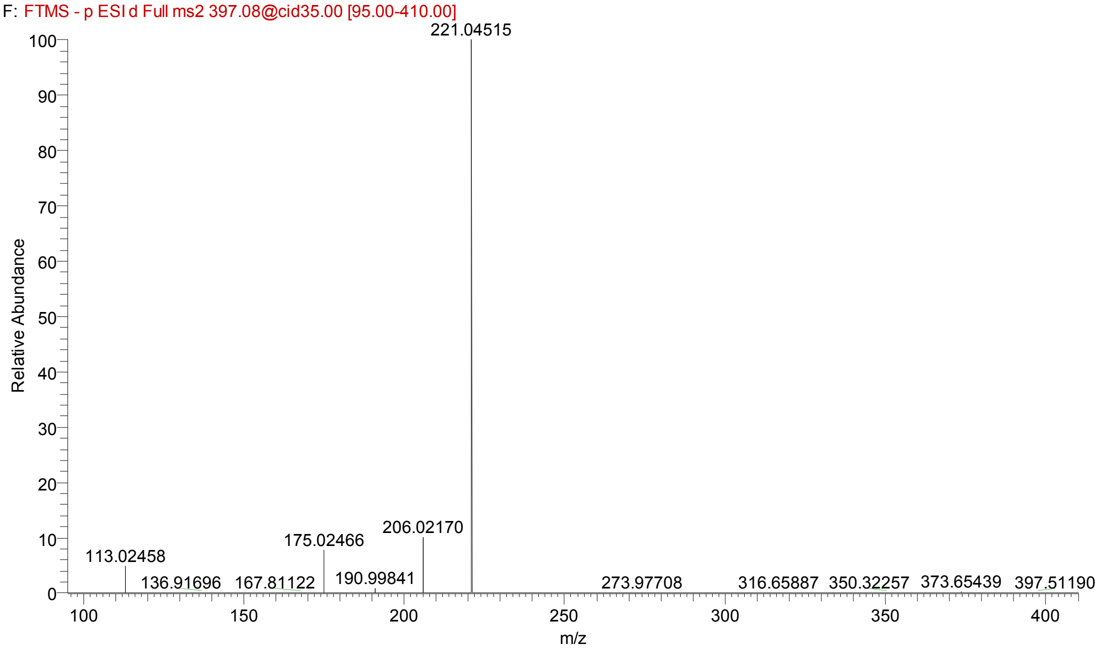

HR-ESI-MS/MS of **101** (Product ion of m/z 397)
