# Supplementary material for: Systematic Analysis of Absorbed Anti-Inflammatory Constituents and Metabolites of Sarcandra glabra in Rat Plasma Using Ultra-High-Pressure Liquid Chromatography Coupled with Linear Trap Quadrupole Orbitrap Mass Spectrometry
Source: PLoS One. 2016 Mar 14;11(3):e0150063. doi: 10.1371/journal.pone.0150063 (PMC4790918; doi:10.1371/journal.pone.0150063)
Supplement: S2 File — (PDF) [file pone.0150063.s002.pdf]

UHPLC Chromatograms of the standards identified in *S. glabra*

1. Quinic acid (t_R=3.7 min, **1**)

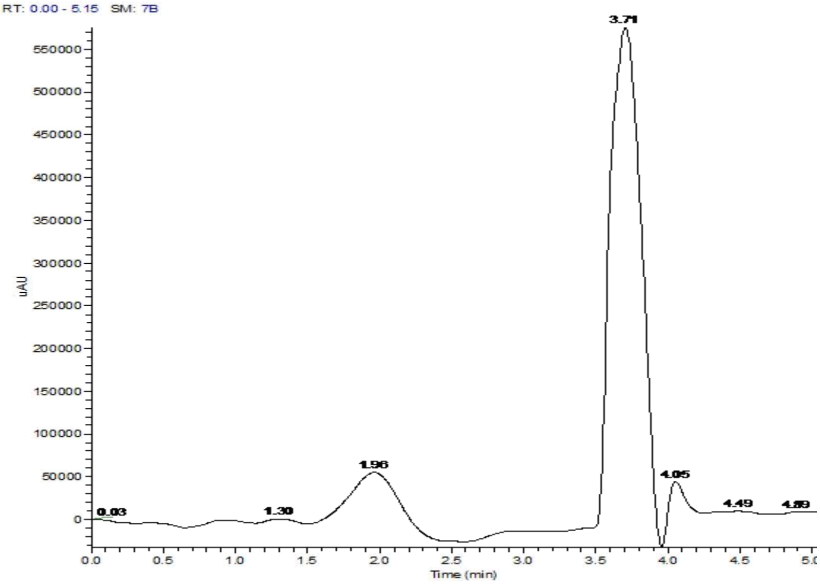

UHPLC Chromatogram of **1**

2, shikimic acid (t_R=3.8 min, **2**)

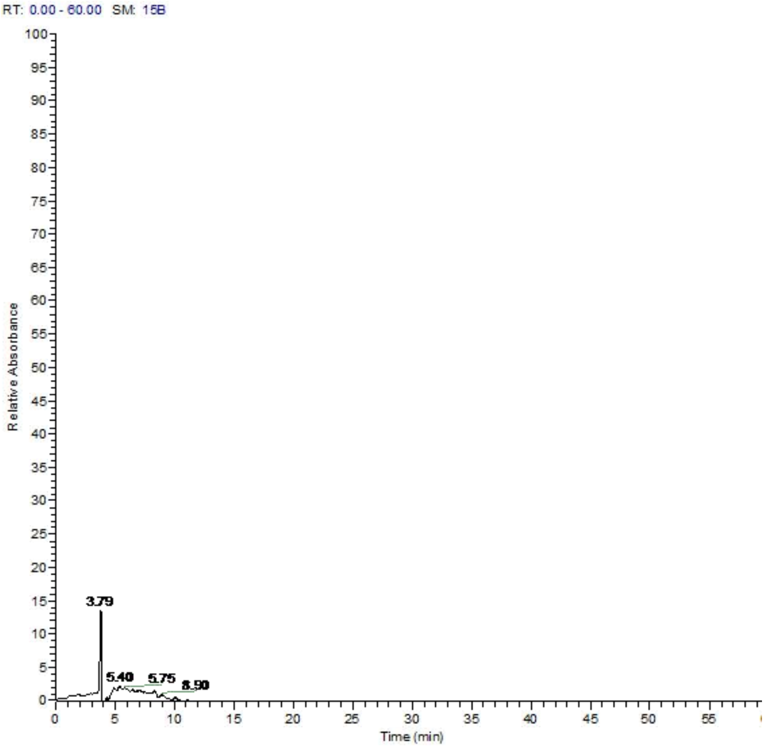

UHPLC Chromatogram of **2**

3, fumaric acid (t_R=6.2 min, **4**)

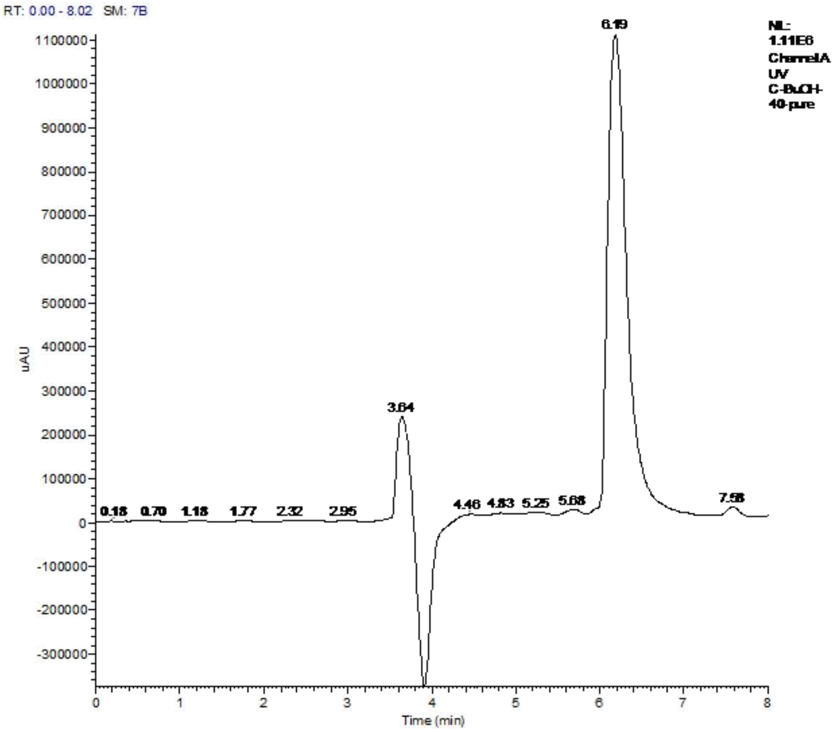

UHPLC Chromatogram of **4**

4, protocatechuic acid (t_R=12.3 min, **11**)

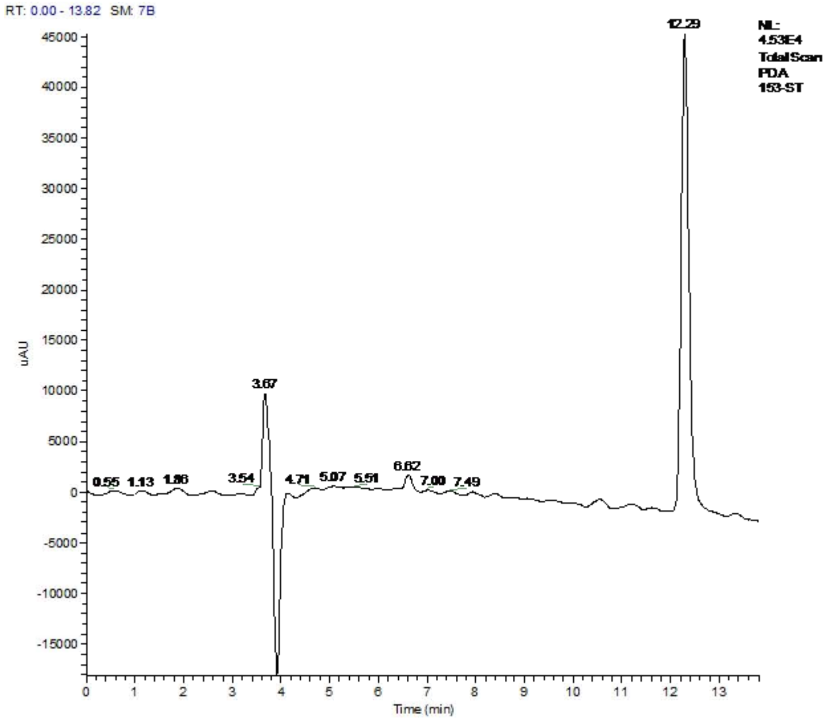

UHPLC Chromatogram of **11**

5, 3-*O*-caffeoylquinic acid (t_R=12.6 min, **12**)

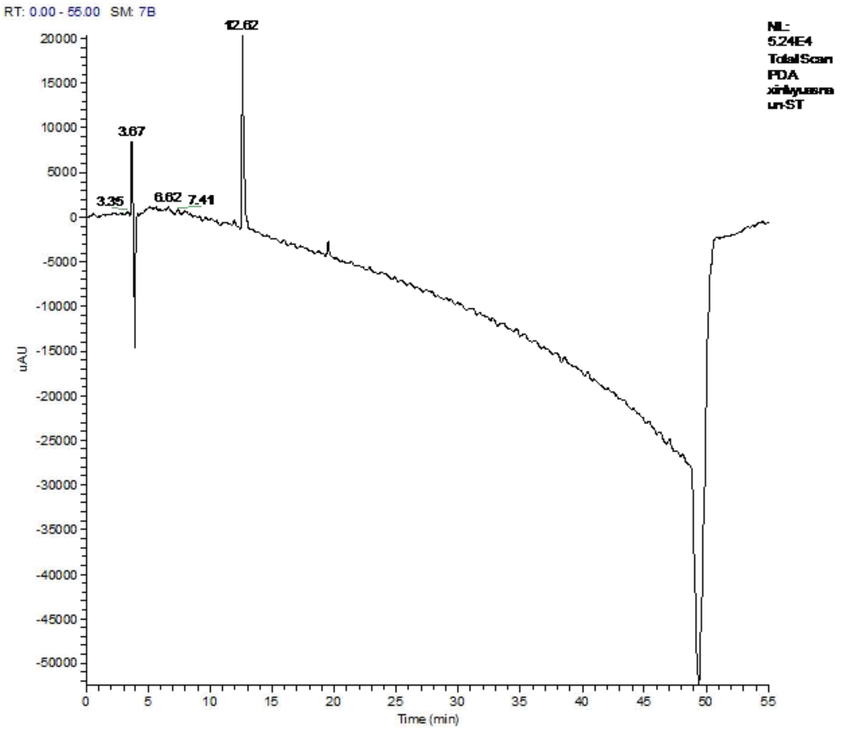

UHPLC Chromatogram of **12**

6, 5-*O*-caffeoylquinic acid (t_R=18.0 min, **18**)

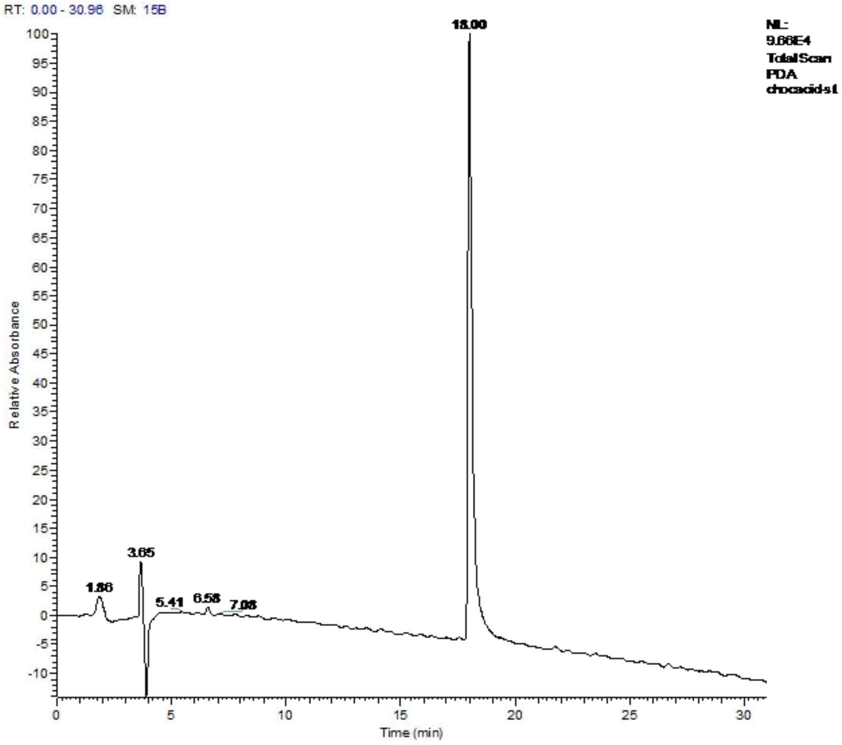

UHPLC Chromatogram of **18**

7, 4-*O*-caffeoylquinic acid (t_R=18.5 min, **19**)

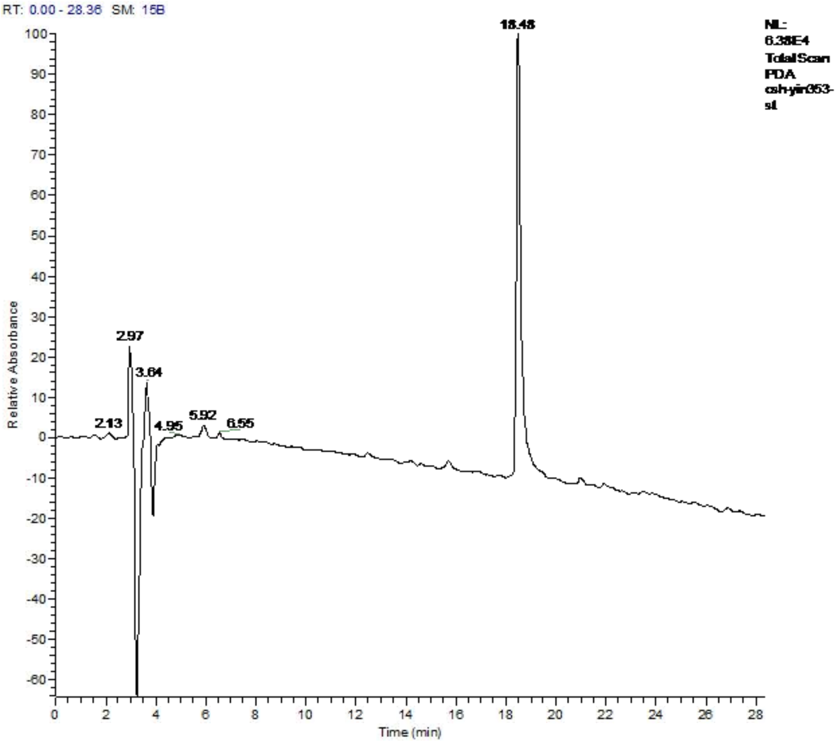

UHPLC Chromatogram of **19**

8, eleutherosideB$_1$ (t_R=19.4 min, **21**)

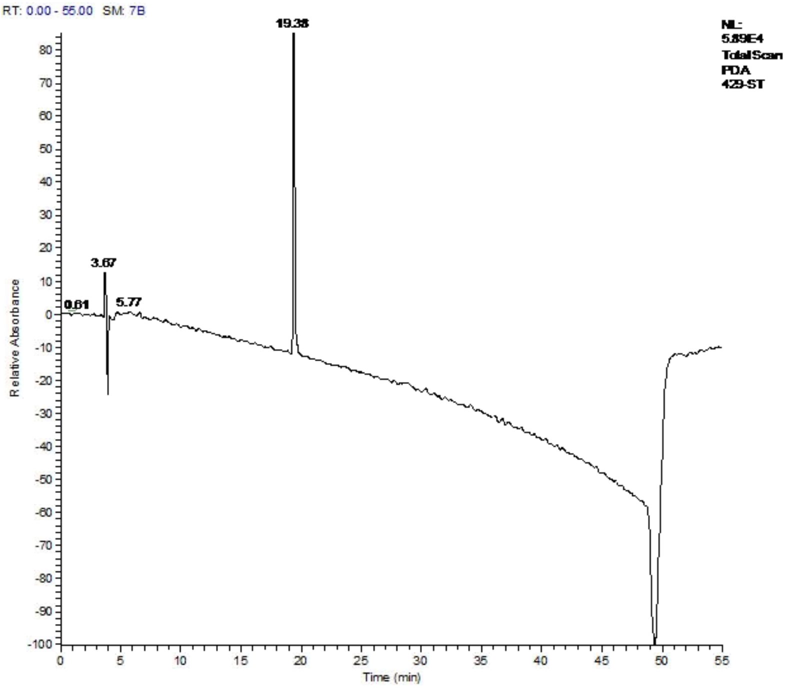

UHPLC Chromatogram of **21**

9， fraxin (t_R=19.8 min, **22**)

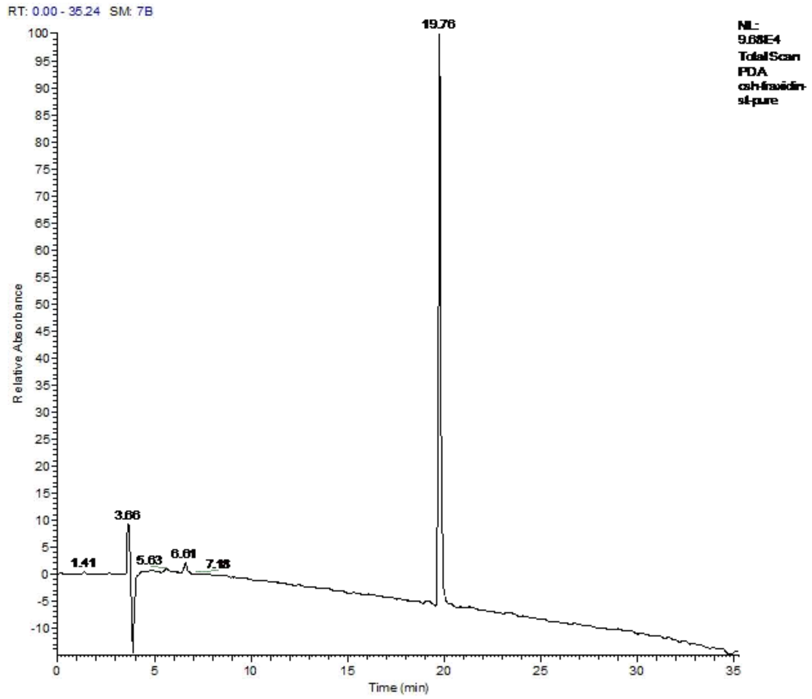

UHPLC Chromatogram of **22**

10, Esculetin (t_R=20.1 min, **24**)

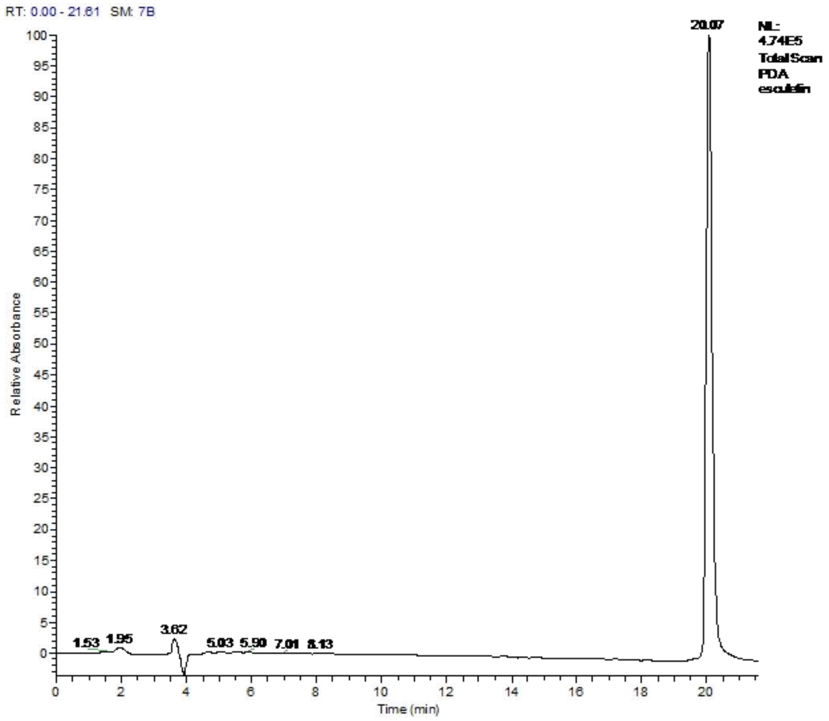

UHPLC Chromatogram of **24**

11，caffeic acid (t_R=20.8 min, **26**)

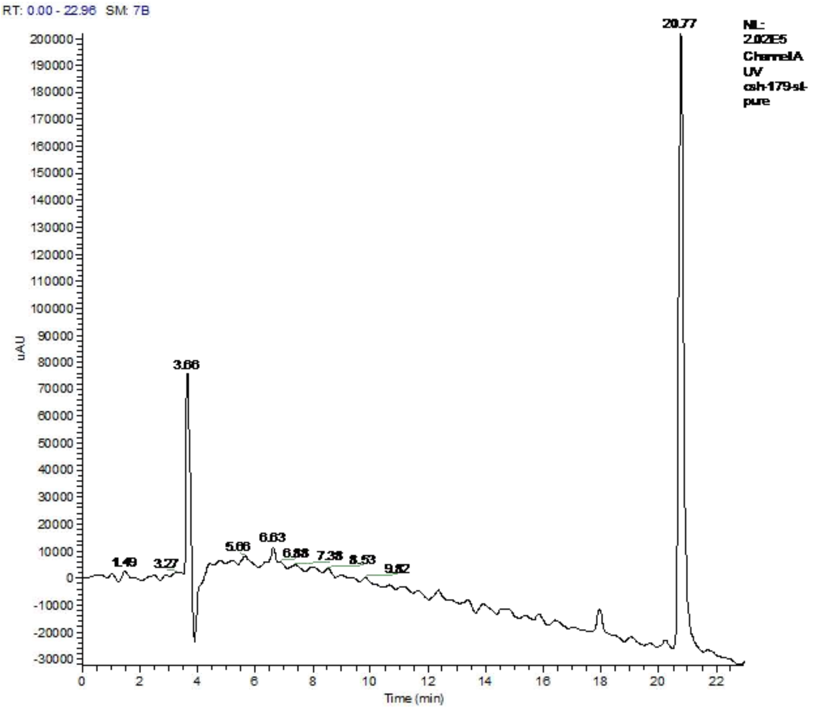

UHPLC Chromatogram of **26**

12, 3-*O*-caffeoylshikimic acid (t_R=21.6 min, **27**) and 4-*O*-caffeoylshikimic acid (t_R=22.2 min, **28**)

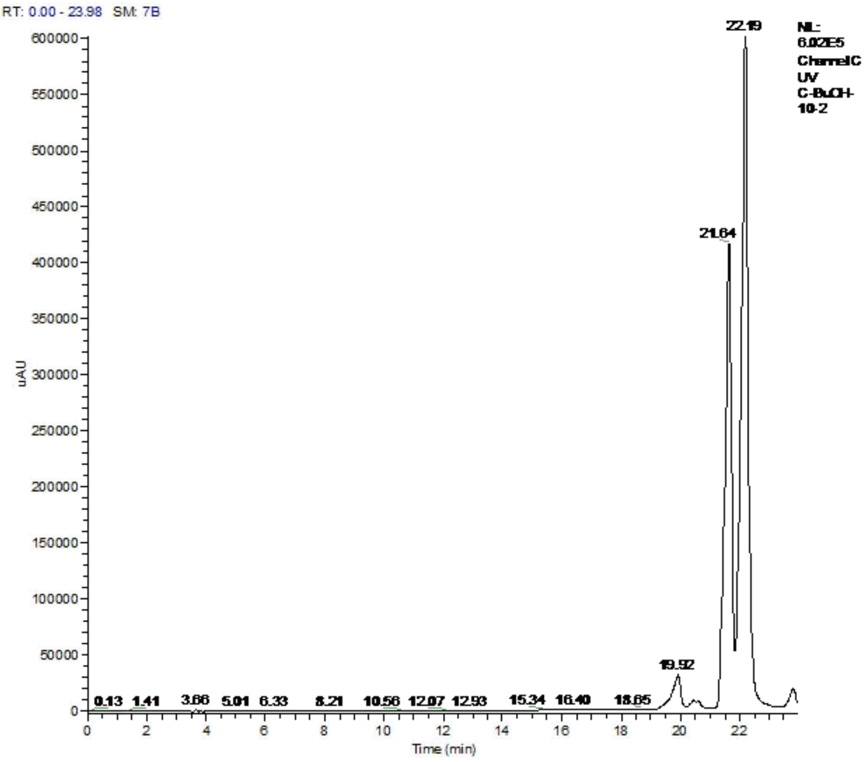

UHPLC Chromatogram of **27** and **28**

13, fraxidin (t_R =23.5 min, **31**)

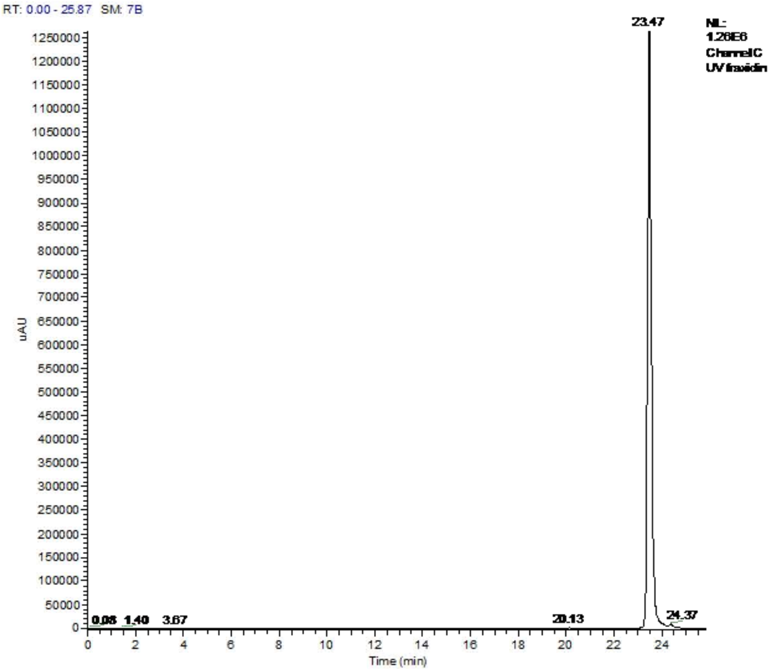

UHPLC Chromatogram of **31**

14, 5-*O*-caffeoylshikimic acid (t_R=23.7 min, **32**)

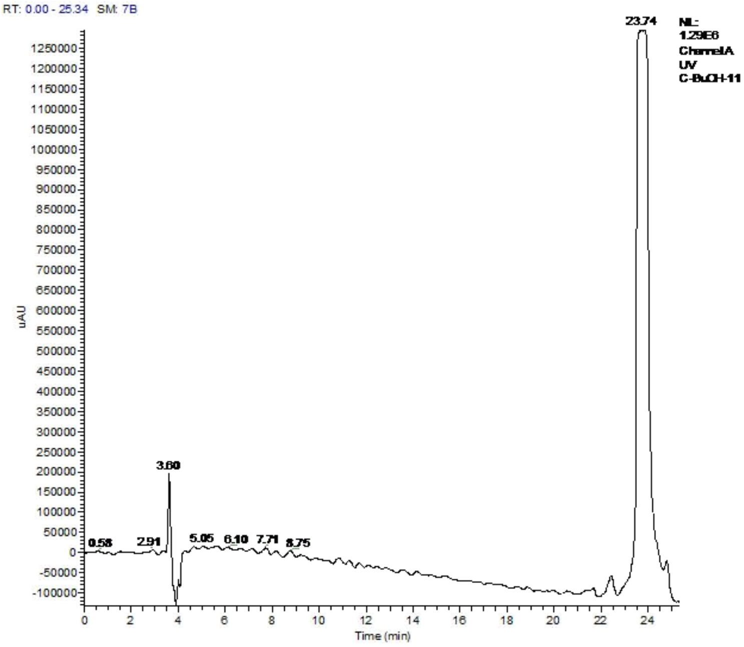

UHPLC Chromatogram of **31**

15，drovomifoliol-*O*-*B*-D-glucopyranoside (t_R=24.0 min, **33**)

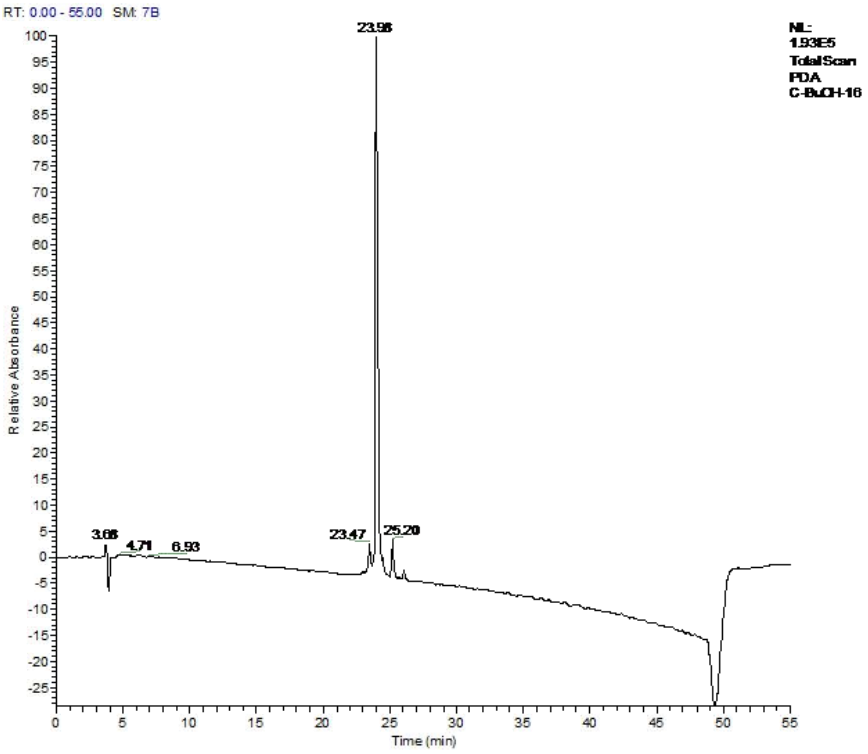

UHPLC Chromatogram of **33**

16, dihydrovomifoliol-*O*-*B*-D-glucopyranoside (t_R=25.2 min, **35**)

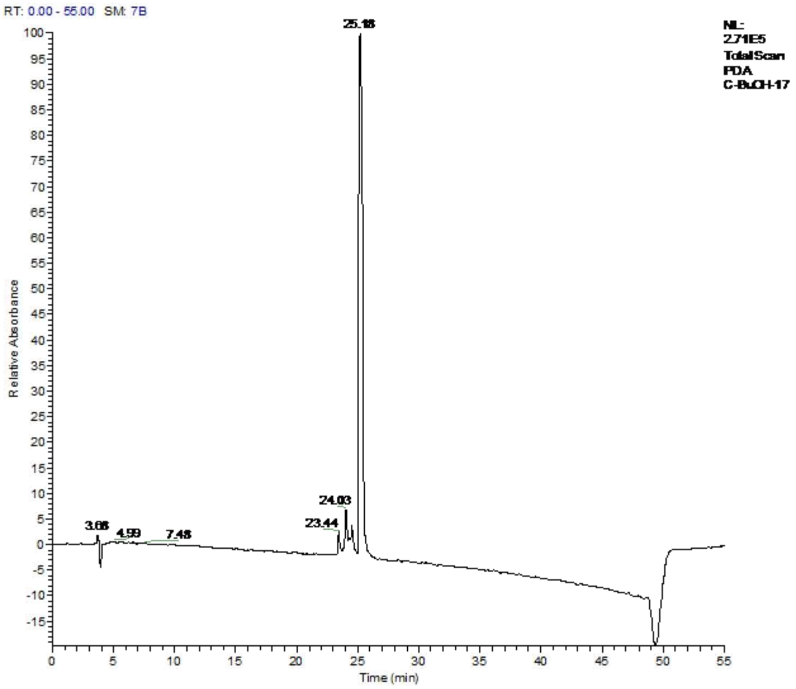

UHPLC Chromatogram of **35**

17, (2R)-naringenin-6-C-*B*-D-glucopyranoside (t_R=25.3 min, **36**) and (2*S*)-naringenin-6- C-*B*-D-glucopyranoside (t_R=26.3 min, **39**)

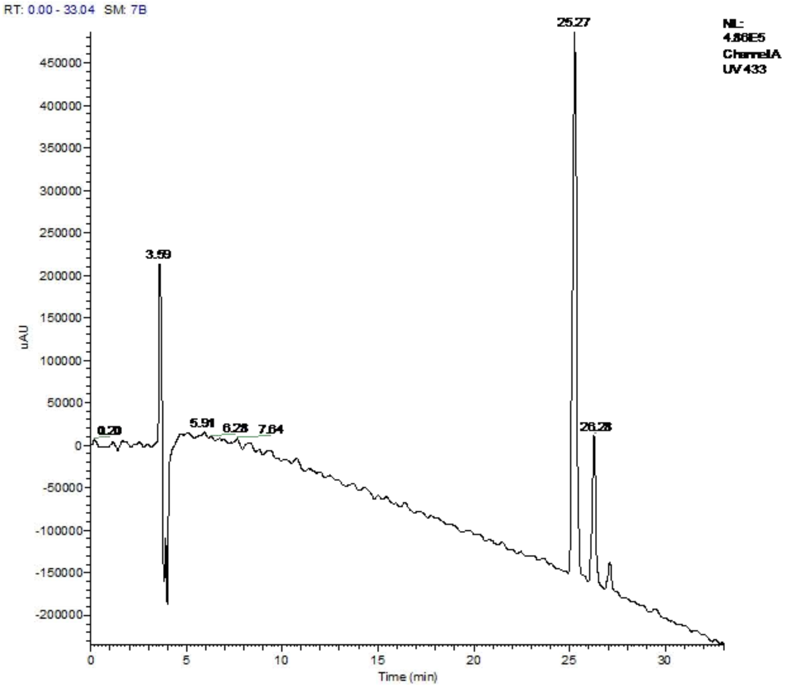

UHPLC Chromatogram of **36** and **39**

18, (2*R*)-naringenin-6-C-*B*-D- glucopyranosyl-(6→1)-apiose (t_R=25.9 min, **37**) and (2*S*)-naringenin-6-C-*B*-D- glucopyranosyl-(6→1)-apiose (t_R=27.2min, **42**)

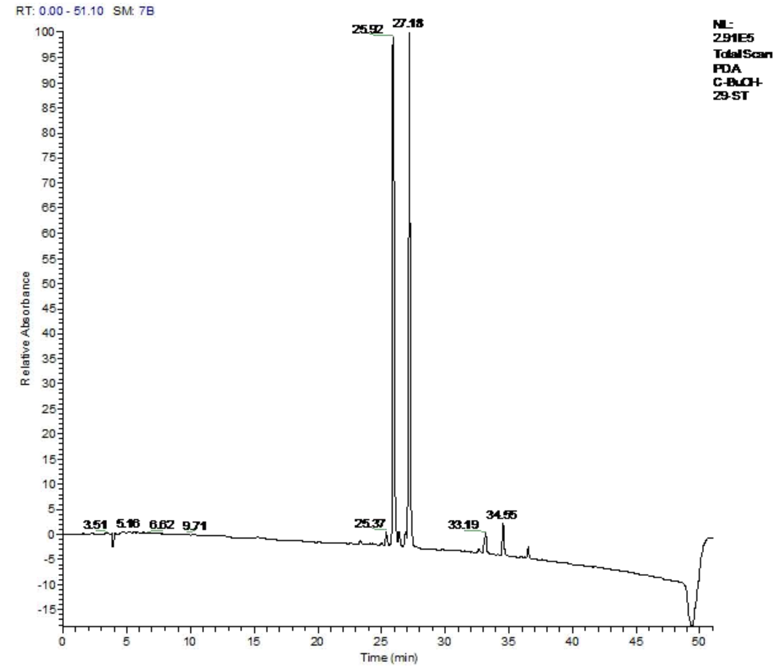

UHPLC Chromatogram of **37** and **42**

19，sarcaglaboside G (t_R=26.1 min, **38**)

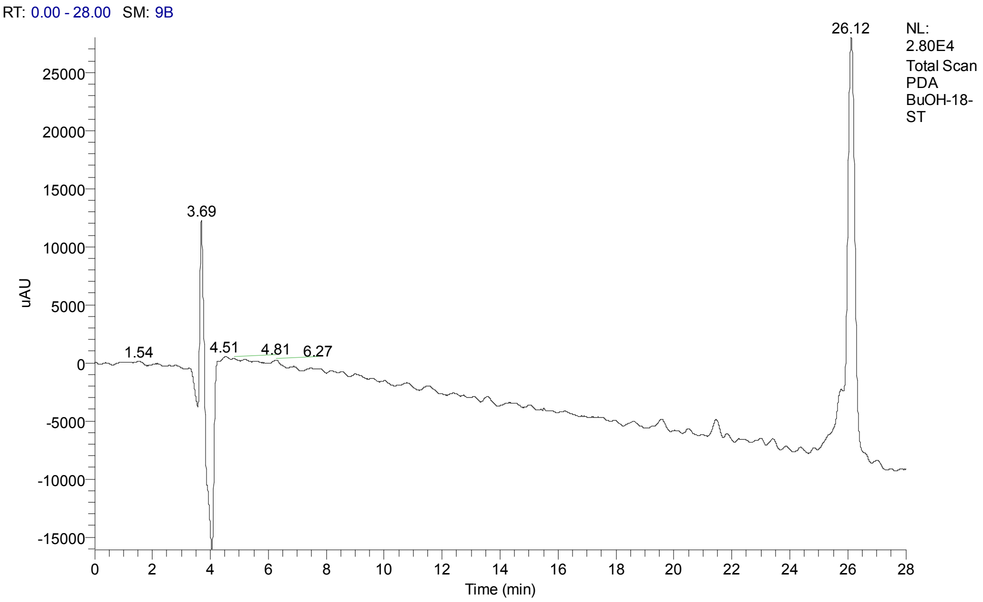

UHPLC Chromatogram of **38**

20, taxifolin (t_R=27.0 min, **41**)

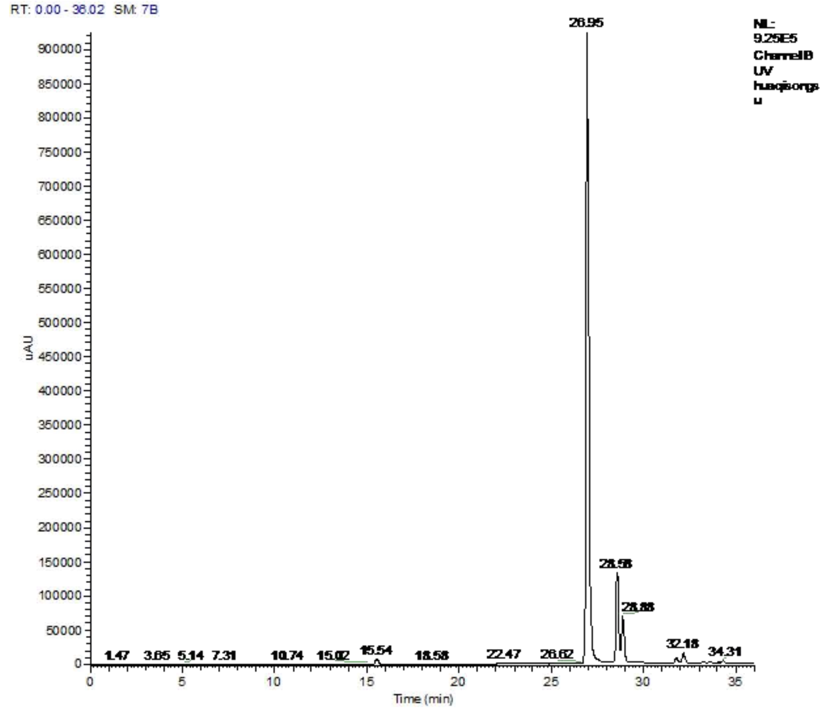

UHPLC Chromatogram of **41**

21, 4,5-dihydroxy-7-rhamnyl-2H-chromen-2-one (t_R=27.4 min, **44**)

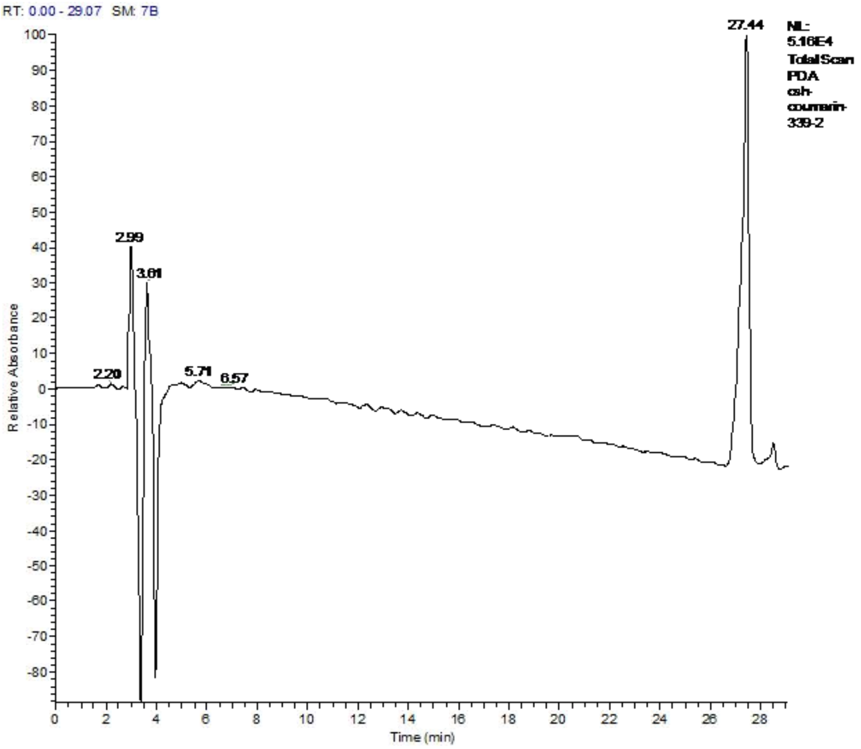

UHPLC Chromatogram of **44**

22, Isofraxidin (t_R=27.9 min, **45**)

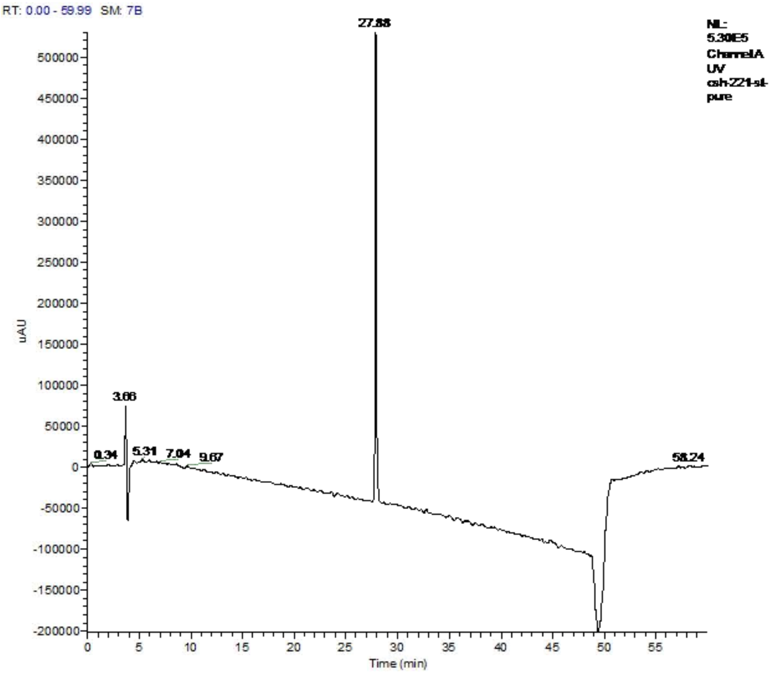

UHPLC Chromatogram of **45**

23, neoastilbin (t_R=29.6 min, **52**)

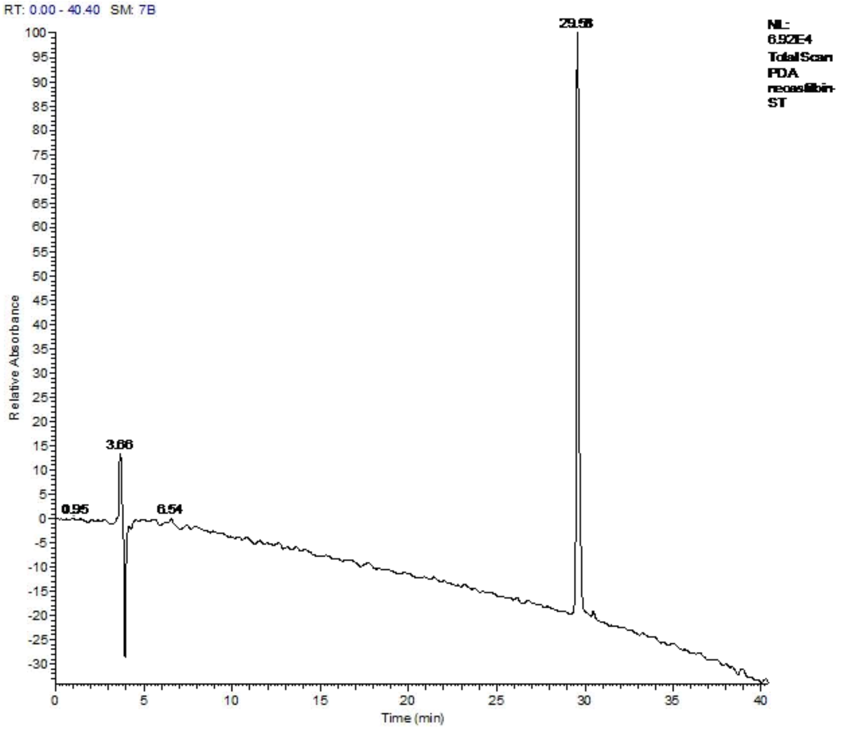

UHPLC Chromatogram of **52**

24, 3, 5-caffeoylquinic acid (t_R=30.2 min, **53**)

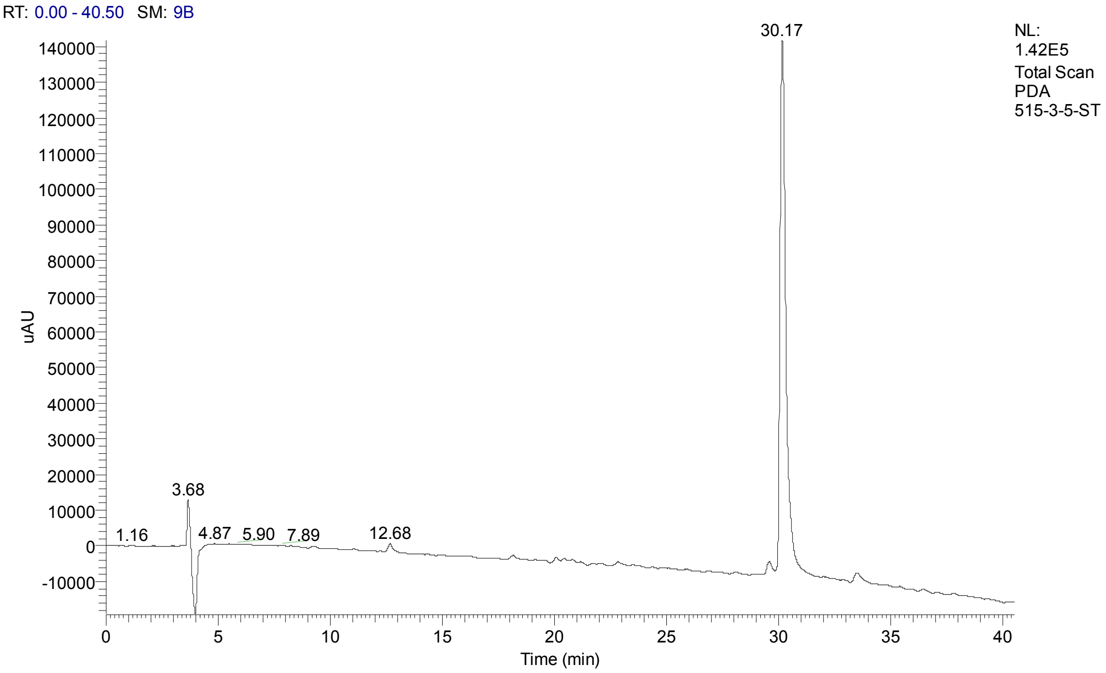

UHPLC Chromatogram of **53**

25, astilbin (t_R=30.7 min, **54**)

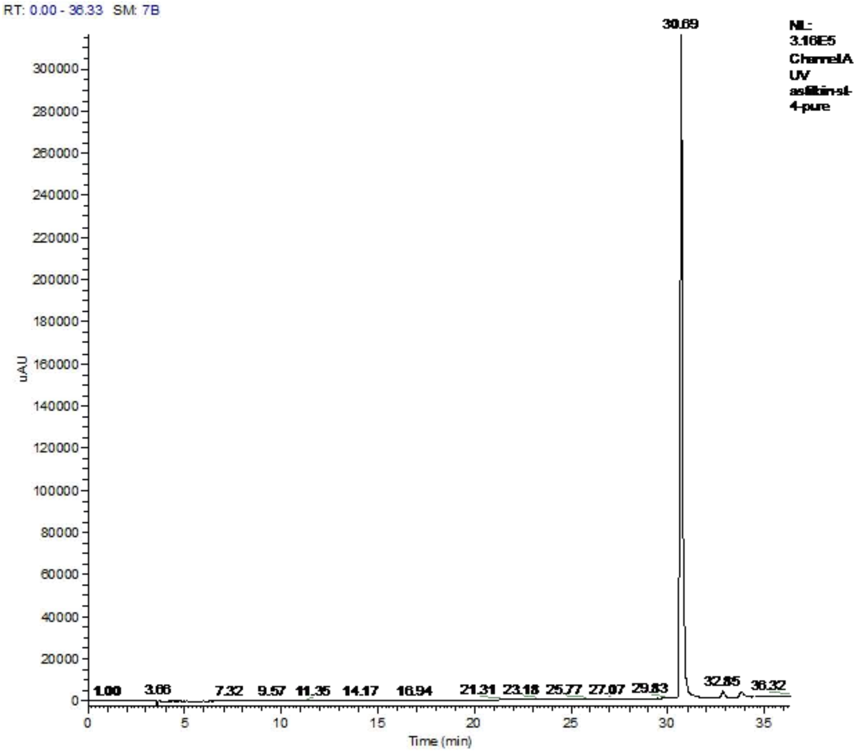

UHPLC Chromatogram of **54**

26，Chloranoside A (t_R=30.9 min, **55**)

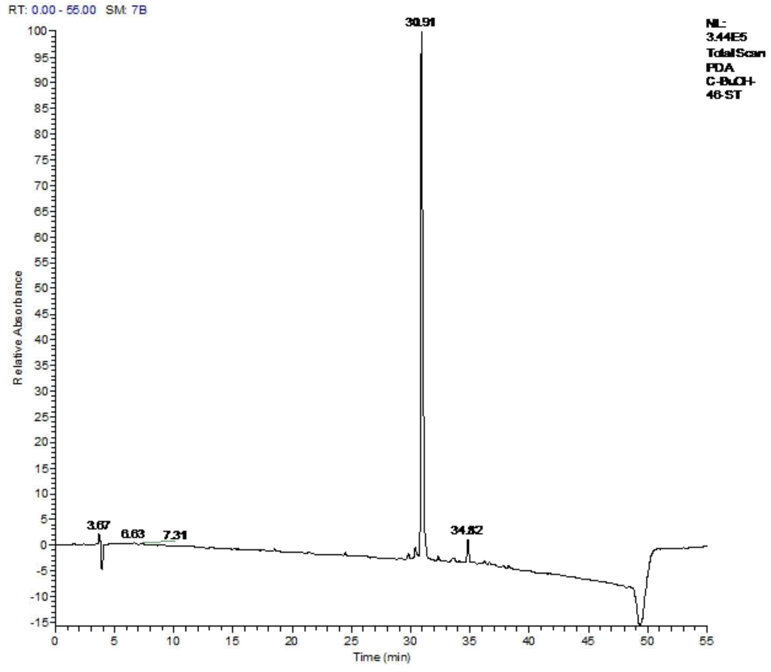

UHPLC Chromatogram of **55**

27, Rosmarinic acid-4-*O*-*β*-D-glucoside (t_R=31.2 min, **56**)

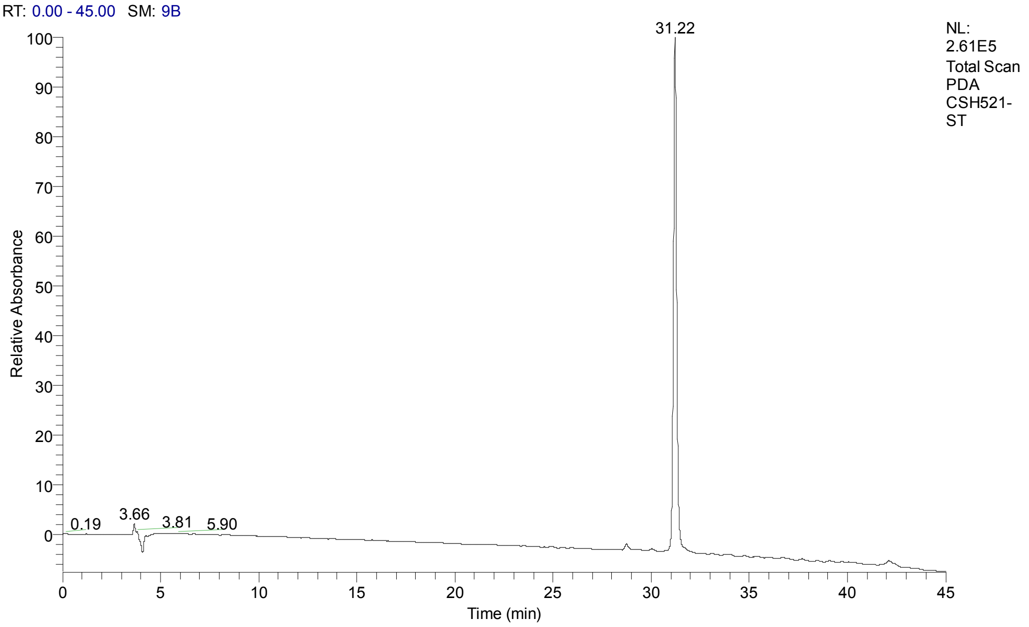

UHPLC Chromatogram of **56**

28, quercetin-3-*O*-*β*-D-glucuronide (t_R=31.7 min, **57**)

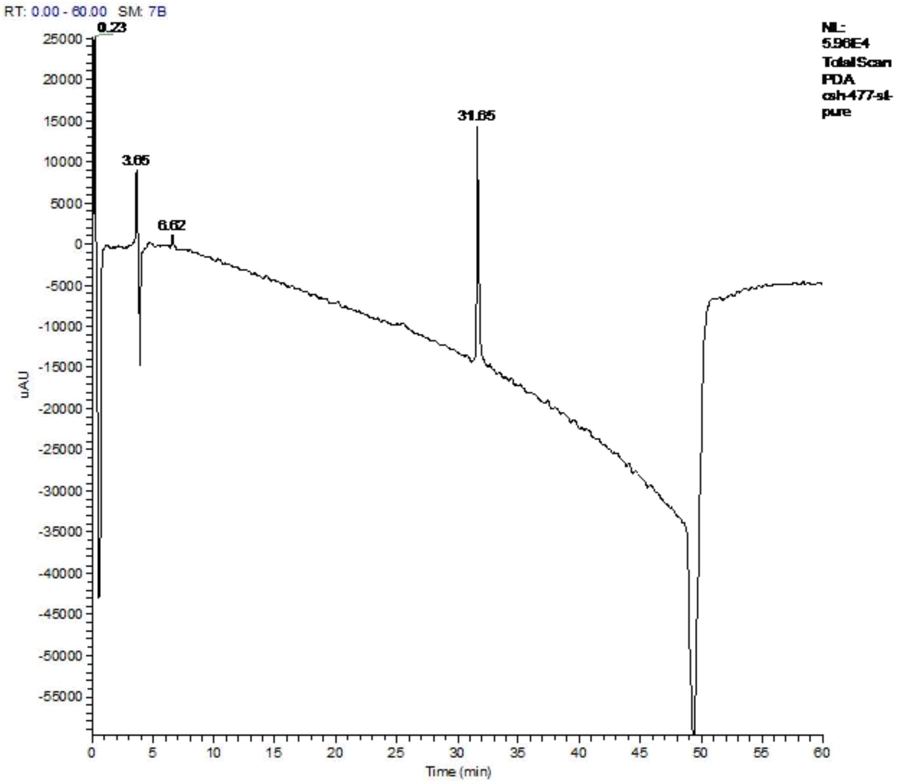

UHPLC Chromatogram of **57**

29, 4, 5-dicaffeoylquinic acid (t_R=33.5 min, **60**)

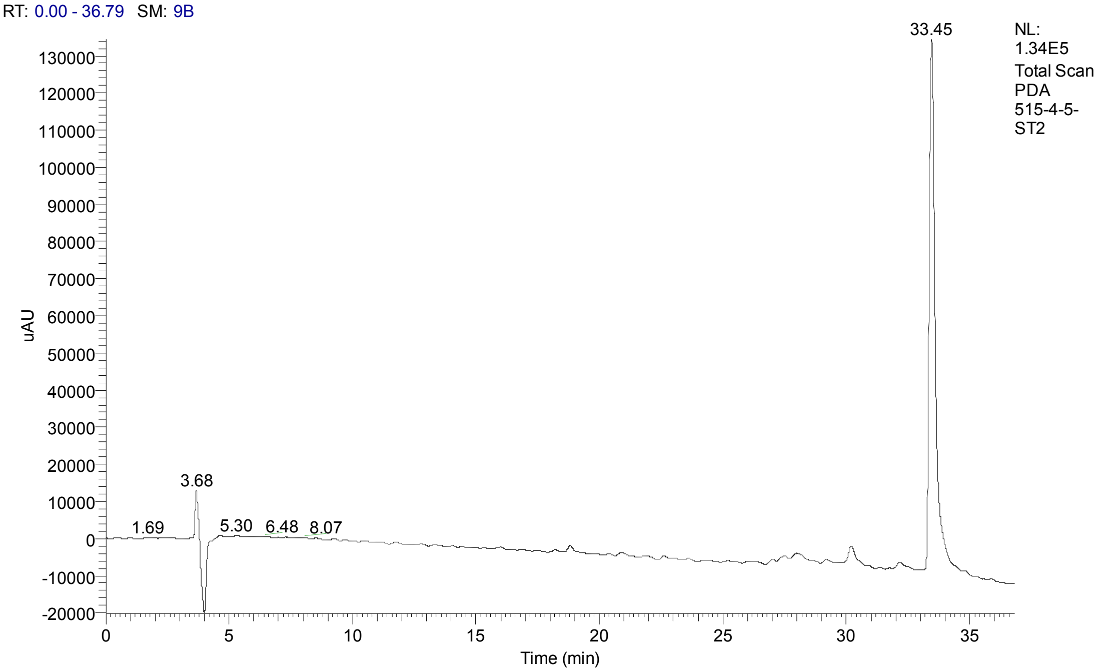

UHPLC Chromatogram of **60**

30，phlorizin (t_R=33.5 min, **61**)

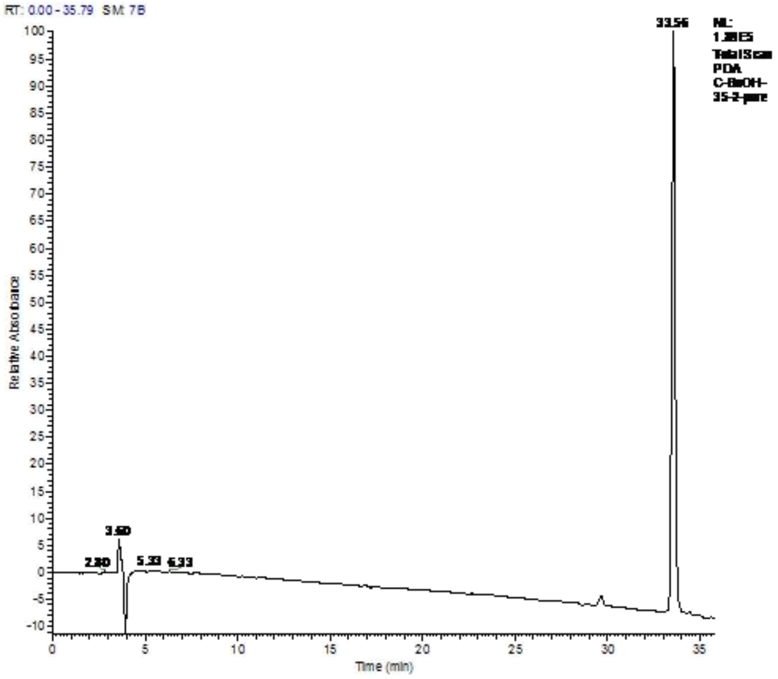

UHPLC Chromatogram of **61**

31, neoisoastilbin (t_R=32.9 min, **62**)

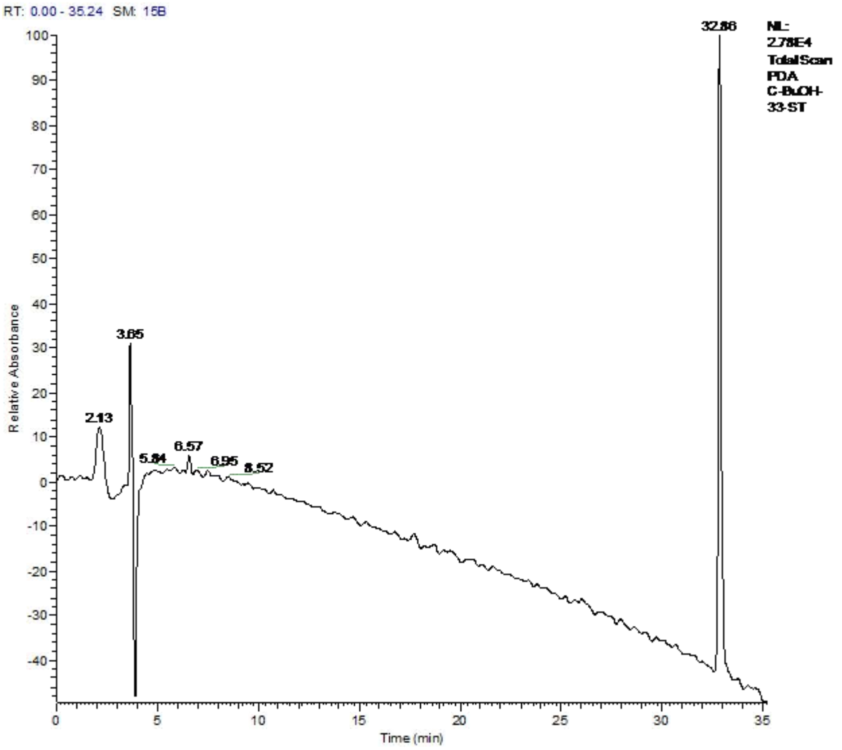

UHPLC Chromatogram of **62**

32， isoastilbin (t_R=33.5 min, **63**)

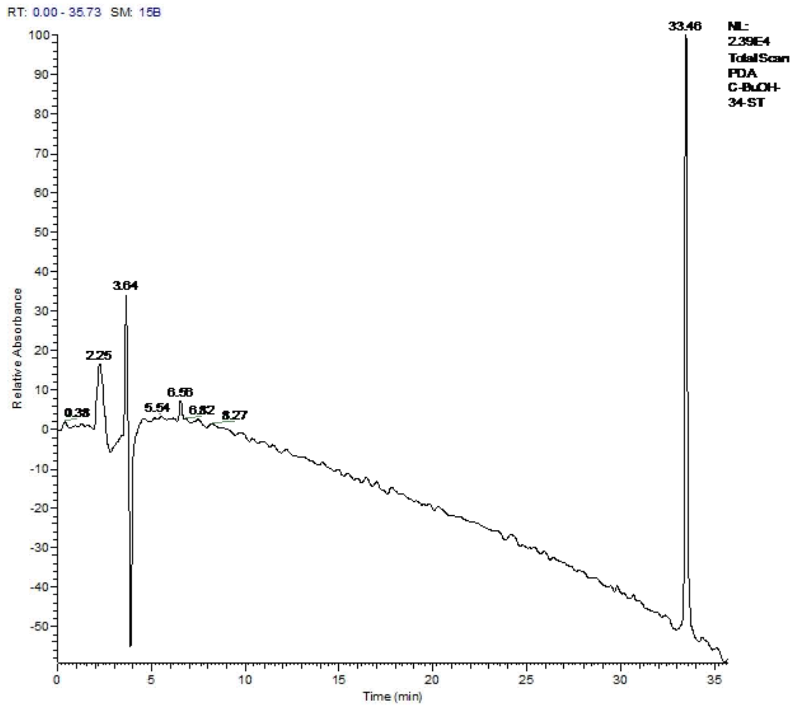

UHPLC Chromatogram of **63**

33, rosmarinic acid (t_R=33.9min, **64**)

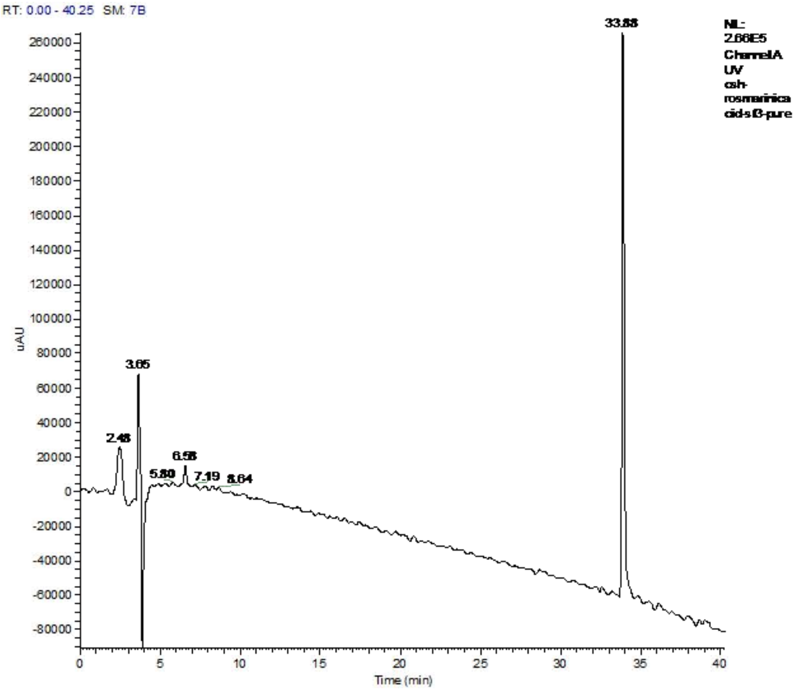

UHPLC Chromatogram of **64**

34，quercitrin (t_R=36.4 min, **67**)

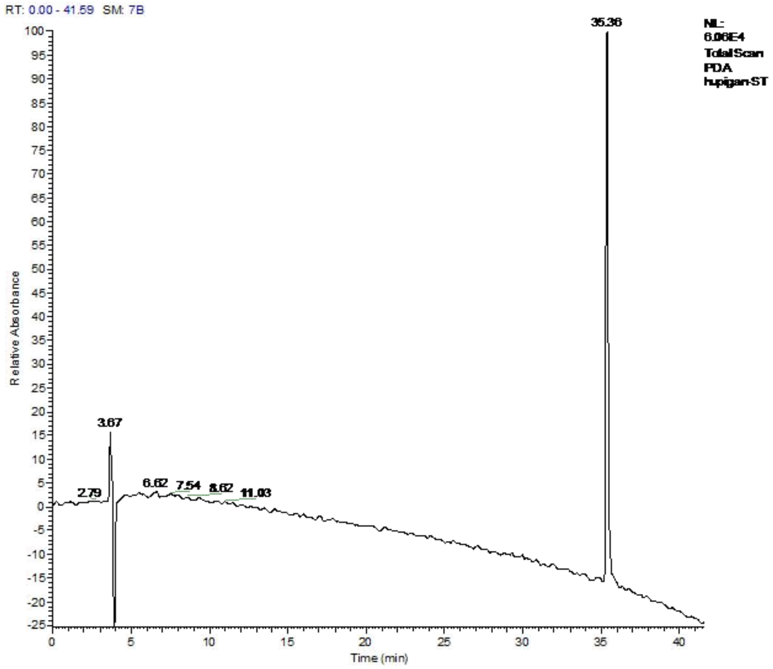

UHPLC Chromatogram of **67**

35，Sarcaboside A (t_R=36.9 min, **73**)

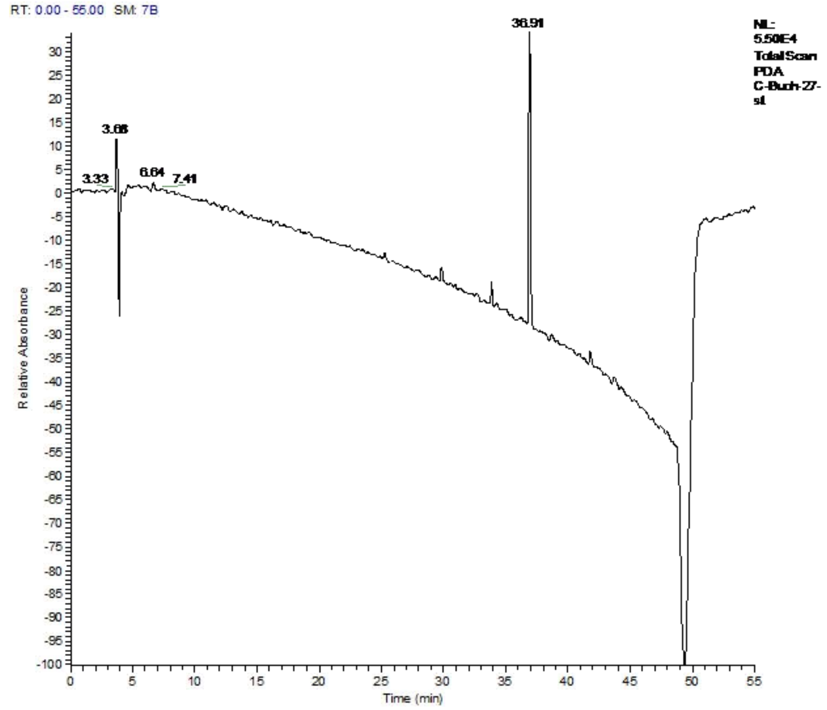

UHPLC Chromatogram of **73**

36，vinyl caffeate (t_R=42.3 min, **82**)

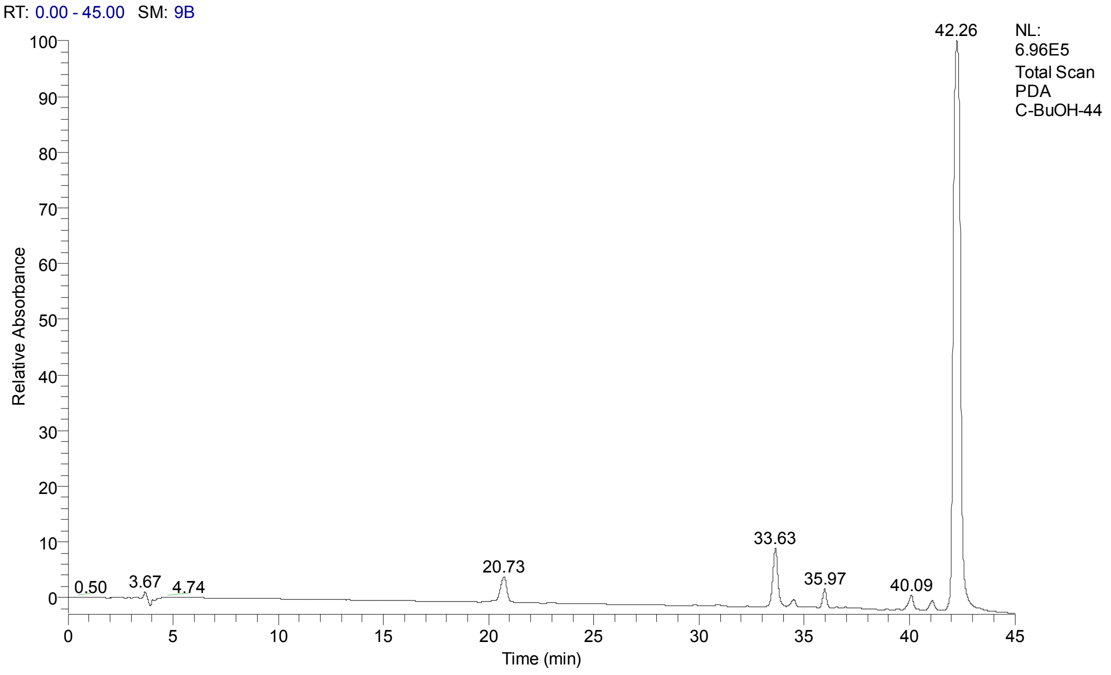

UHPLC Chromatogram of **82**

37，8*β*,9*α*-dihydroxylindan-(5),7(1)-ieb-8α, 12-olide (t_R=43.3 min, **86**)

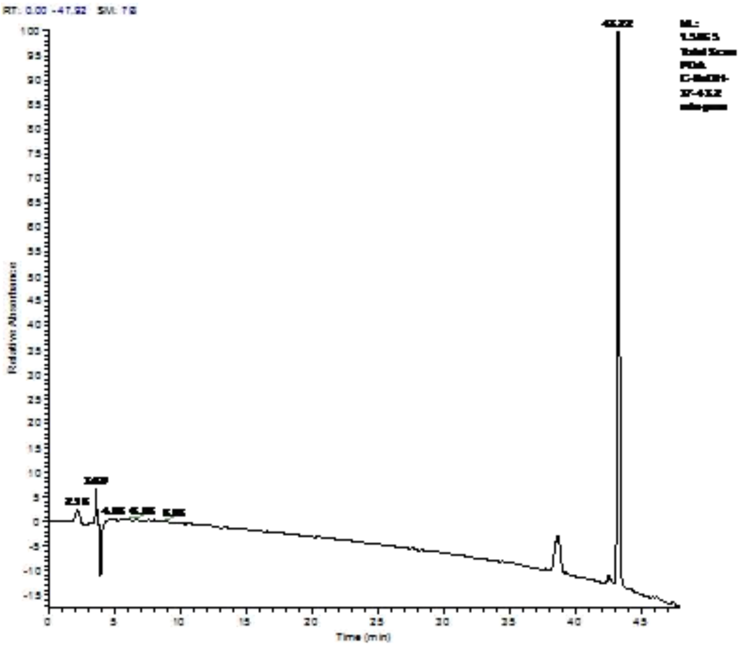

UHPLC Chromatogram of **86**

38, 5-hydroxy-7,8-dimethoxy-flavanone (t_R=44.4min, **88**)

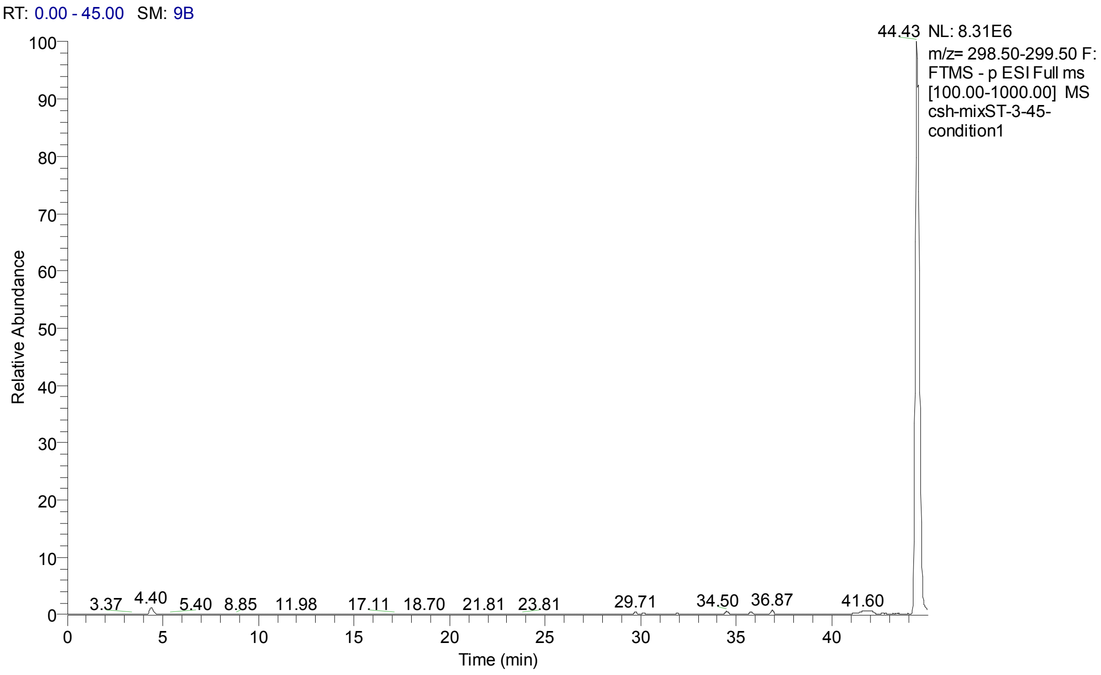

UHPLC Chromatogram of **88**

39, Chloranthalactone E (t_R=47.6 min, **89**)

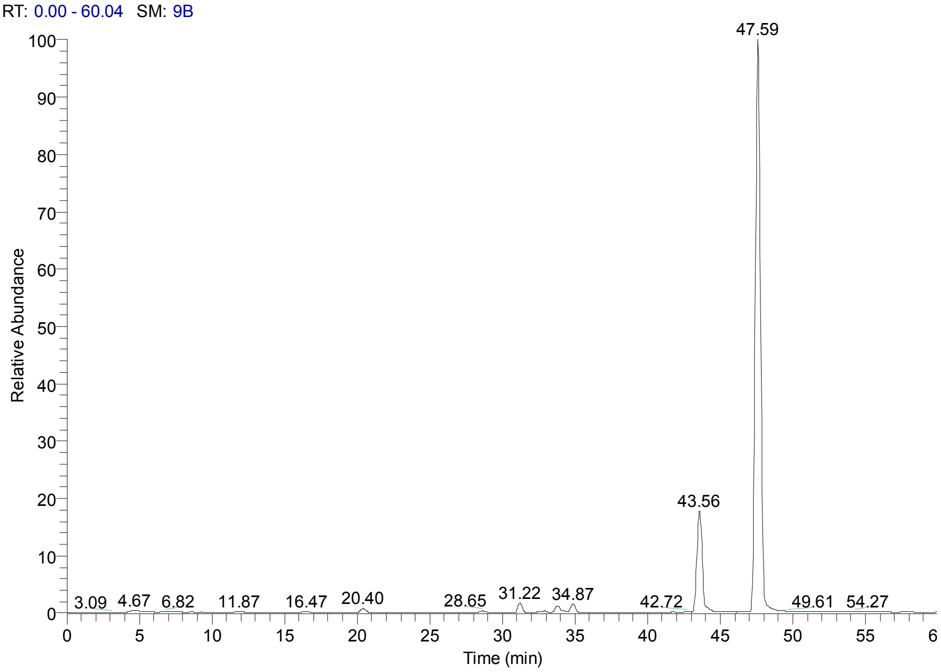

UHPLC Chromatogram of **89**

40, *p*-coumaric acid (t_R=27.0 min, **98**)

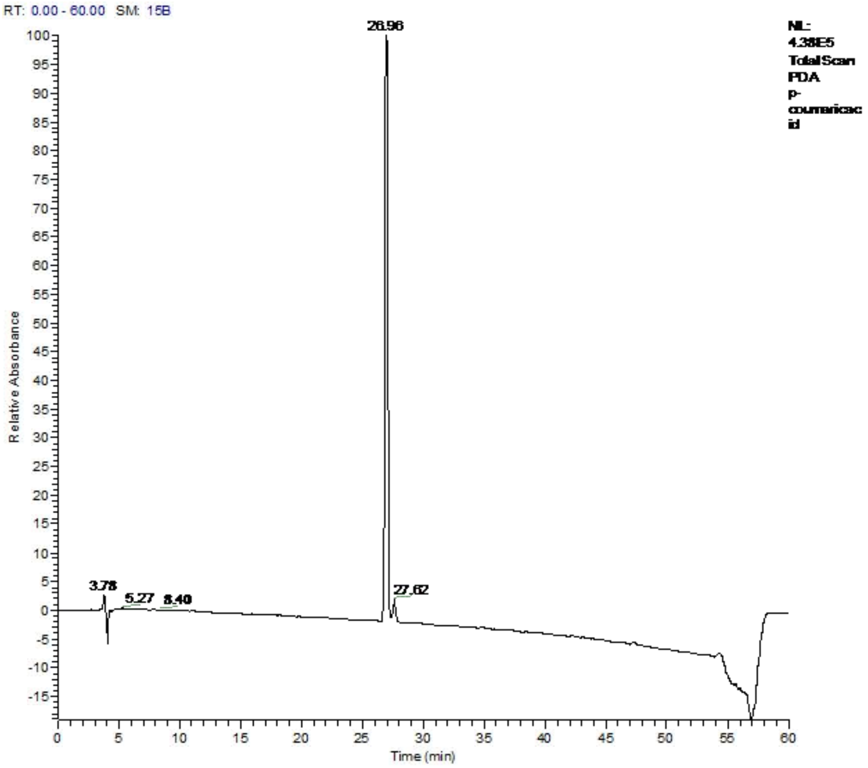

UHPLC Chromatogram of **98**

41, isofraxidin -7-O-sulphate (t_R=18.9 min, **100**)

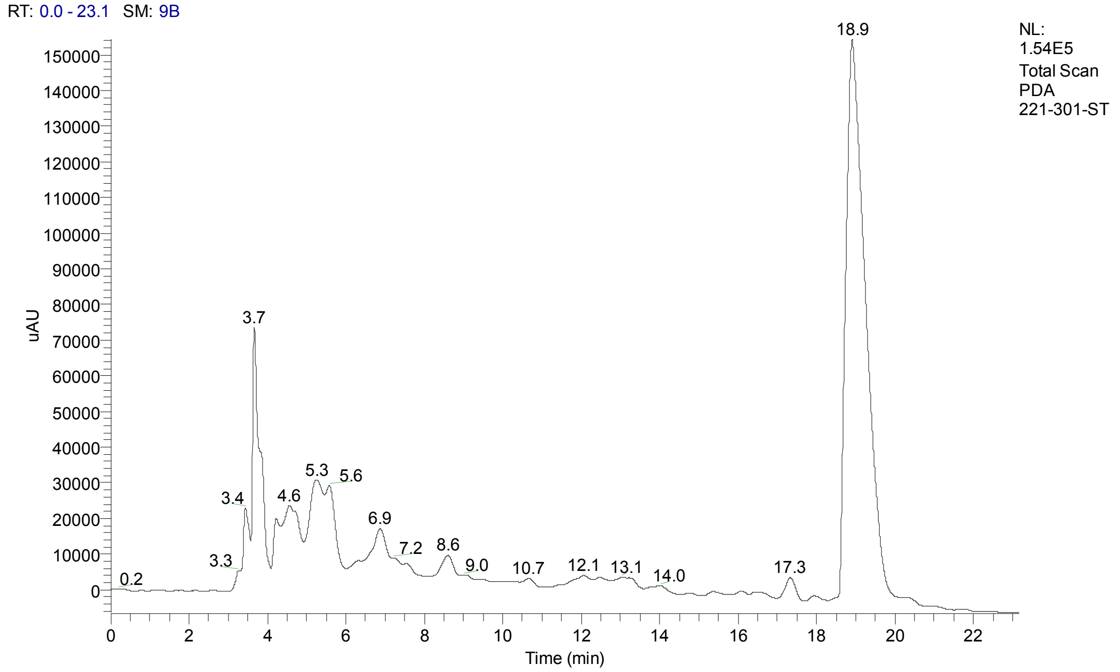

UHPLC Chromatogram of **100**

42, isofraxidin -7-O-α-D-glucuronide (t_R=19.5 min, **101**)

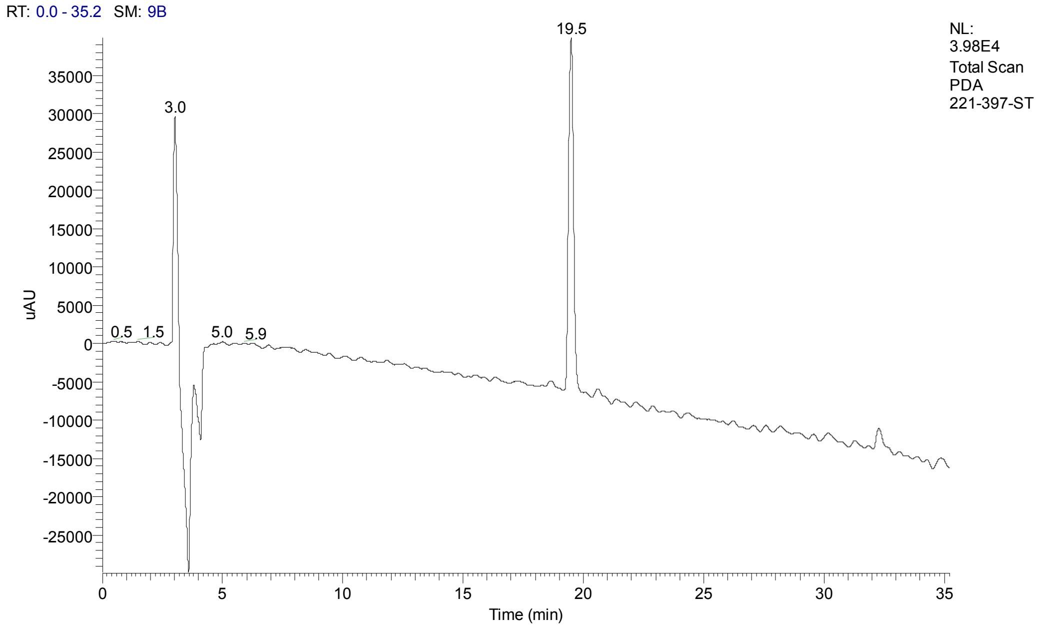

UHPLC Chromatogram of **101**

43, ferulic acid (t_R=28.6min, **136**)

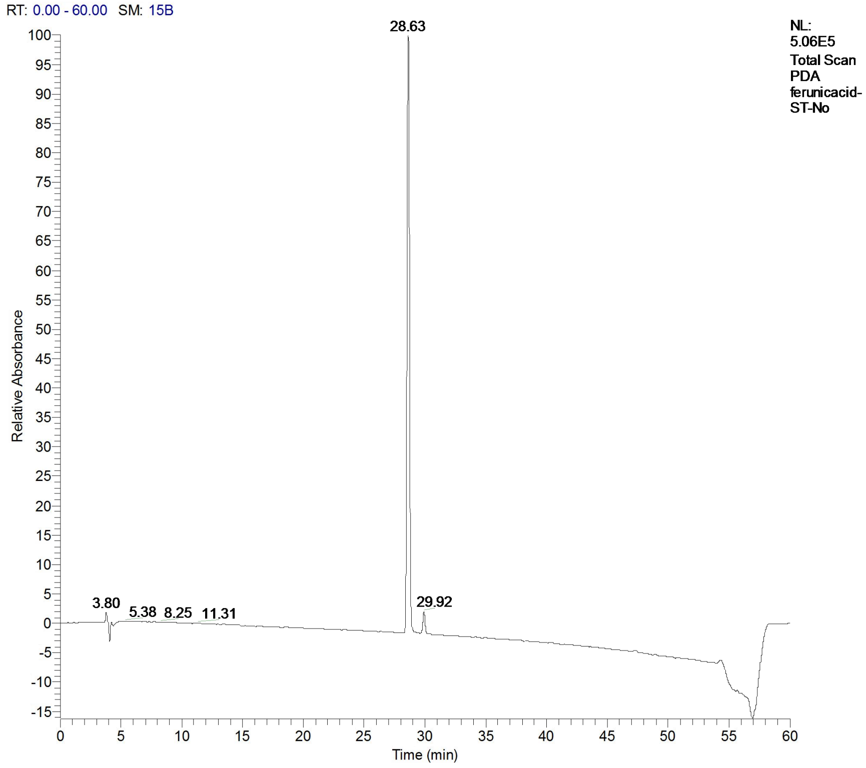

UHPLC Chromatogram of **136**

44, isoferulic acid (t_R=30.3 min, **135**)

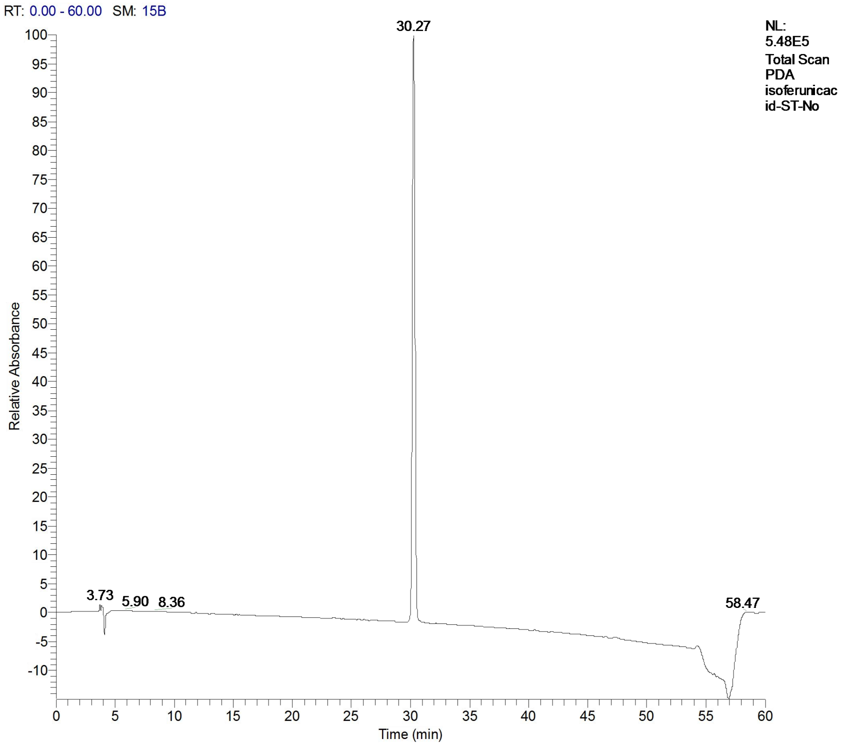

UHPLC Chromatogram of **135**
