# Supplementary material for: Systematic Analysis of Absorbed Anti-Inflammatory Constituents and Metabolites of Sarcandra glabra in Rat Plasma Using Ultra-High-Pressure Liquid Chromatography Coupled with Linear Trap Quadrupole Orbitrap Mass Spectrometry
Source: PLoS One. 2016 Mar 14;11(3):e0150063. doi: 10.1371/journal.pone.0150063 (PMC4790918; doi:10.1371/journal.pone.0150063)
Supplement: S1 Table — (PDF) [file pone.0150063.s003.pdf]

S1 Table. Sensitivity evaluation of representative constituents from *S. glabra*

Compound	Category	C (ng/ml)	S/N	Detecting Mode
3-*O*-caffeoylquinic acid (**12**)	caffeoylquinic acids	500	2015	non-target FT-FT
5-*O*-caffeoylquinic acid (**18**)	caffeoylquinic acids	500	1248	non-target FT-FT
4-*O*-caffeoylquinic acid (**19**)	caffeoylquinic acids	500	1190	non-target FT-FT
eleutheroside B_1(**21**)	coumarins	500	2062	non-target FT-FT
Fraxin (**22**)	coumarins	500	2956	non-target FT-FT
Isofraxidin (**45**)	coumarins	500	10232	non-target FT-FT
neoastilbin (**52**)	flavonoids	500	1983	non-target FT-FT
Astilbin (**54**)	flavonoids	500	1181	non-target FT-FT
Rosmarinic acid-4-*O*-*β*-D-glucoside (**56**)	dicaffeoyl derivatives	500	1488	non-target FT-FT
quercetin-3-*O*-*β*-D-glucuronide (**57**)	flavonoids	500	25755	non-target FT-FT
neoisoastilbin(**62**)	flavonoids	500	1108	non-target FT-FT
Isoastilbin (**63**)	flavonoids	500	786	non-target FT-FT
Rosmarinic acid (**64**)	dicaffeoyl derivatives	500	9482	non-target FT-FT
quercitrin (**67**)	flavonoids	500	1627	non-target FT-FT
5-*O*-caffeoylquinic acid (**18**)	caffeoylquinic acids	5	7	Target IT-IT
eleutheroside B_1 (**21**)	coumarins	5	10	Target IT-IT
caffeic acid (**26**)	phenolic acids	5	40	Target IT-IT
Astilbin (**54**)	flavonoids	5	12	Target IT-IT
neoisoastilbin(**62**)	flavonoids	5	15	Target IT-IT
protocatechuic acid (**11**)	phenolic acids	1	11	Target IT-IT
3-*O*-caffeoylquinic acid (**12**)	caffeoylquinic acids	0.5	13	Target IT-IT
fraxin (**22**)	coumarins	0.5	24	Target IT-IT

Isofraxidin (**45**)	coumarins	0.5	23	Target IT-IT
Rosmarinic acid-4-*O*-*β*-D-glucoside (**56**)	dicaffeoyl derivatives	0.5	56	Target IT-IT
quercetin-3-*O*-*β*-D-glucuronide (**57**)	flavonoids	0.5	100	Target IT-IT
Rosmarinic acid (**64**)	dicaffeoyl derivatives	0.5	18	Target IT-IT
quercitrin (**67**)	flavonoids	0.5	79	Target IT-IT
Chloranthalactone E (**89**)	sesquiterpene	0.5	15	Target IT-IT
