# Supplementary material for: Systematic Analysis of Absorbed Anti-Inflammatory Constituents and Metabolites of Sarcandra glabra in Rat Plasma Using Ultra-High-Pressure Liquid Chromatography Coupled with Linear Trap Quadrupole Orbitrap Mass Spectrometry
Source: PLoS One. 2016 Mar 14;11(3):e0150063. doi: 10.1371/journal.pone.0150063 (PMC4790918; doi:10.1371/journal.pone.0150063)
Supplement: S2 Table — (PDF) [file pone.0150063.s004.pdf]

S2 Table. Profiling of constituents in *S.glabra* and the absorbed constituents in rat plasma by UHPLC-LTQ/Oribtrap

No.	t_R (min)	UV λ max (nm)	Observed Mass	Error (ppm)	Formula	LC/MS^n data (% base peak)	Identification	Plasma 2
**1** [a]	3.63	–	191.05620	0.5	$C_7H_{11}O_6$	MS^2[191] 173.00903 (100), 127.04015 (34), 85.02979	quinic acid	√
**2**	3.67		253.00230	-0.2	$C_7H_9O_8S$	MS^2[253]173.04543 (100)	shikimic acid sulfate	
**3** [a]	3.87		173.04567	0.9	$C_7H_9O_5$	MS^2[173] 99.04547 (100)	shikimic acid	
**4** [a]	6.15	211	115.00413	3.9	$C_4H_3O_4$	MS^2[115] 71.01418 (100)	fumaric acid	√
**5**	6.71		161.04579	1.5	$C_6H_9O_5$	MS^2[161] 99.04547 (100)	unknown	
**6**	8.07		455.08646	-0.1	$C_{16}H_{23}O_{13}S$	MS^2[455] 241.00198 (100)	unknown	
**7**	8.89		301.05981	-0.2	$C_9H_{17}O_9S$	MS^2[301] 241.00200 (100), 96.96033 (83), 180.98111(18)	sulfur-containing derivatives	
**8**	9.75		359.09833	-0.1	$C_{15}H_{19}O_{10}$	MS^2[359] 197.04541 (100) ($C_9H_9O_5$), 179.03493 (13)	Phenolic acid derivatives	√
**9**	10.10		315.07190	-0.8	$C_{13}H_{15}O_9$	MS^2[315] 153.01929 (100)	Phenolic acid glycoside	
**10**	10.58		197.04587	0.3	$C_9H_9O_5$	MS^2[197]179.03502	Phenolic acid derivatives	
**11**[a]	12.26	259, 293	153.01956	1.5	$C_7H_5O_4$	MS^2[153] 109.02969 (100)	protocatechuic acid	√
**12**[a]	12.44	220,324	353.08762	-0.5	$C_{16}H_{17}O_9$	MS^2[353] 191.05600 (100), 179.03497 (46), 135.04532 (7)	3-*O*-caffeoylquinic acid	
**13**	14.90		181.05080	0.9	$C_9H_9O_4$	MS^2[181]163.04001(100)	dihydrocaffeic acid	√
**14**	15.56		315.07550	-0.1	$C_{10}H_{19}O_9S$	MS^2[315]241.00253 (100), 180.98154 (22), 96.96062（67）	sulfur-containing derivatives	√
**15**	15.74		137.02472	2.2	$C_7H_5O_3$	MS^2[137] 93.03488	hydroxybenzoic acid deraviative	√
**16**	16.86		611.16162	-0.2	$C_{27}H_{31}O_{16}$	MS^2[611] 491.11832(100), 521.12897,	Unknown	

						401.08707, 371.07648		
**17**	17.00		591.15656	-0.2	$C_{24}H_{31}O_{17}$	MS^2[591] 545.14996 (100), 221.04509	Unknown	
**18**[a]	17.85	220,324	353.08762	-0.5	$C_{16}H_{17}O_9$	MS^2[353]191.0557(100),179.0343(7), 173. 0450 (5)	5-*O*-caffeoylquinic acid	√
**19**[a]	18.51	220,324	353.08755	-0.7	$C_{16}H_{17}O_9$	MS^2[353]191.0549(19),179.0338(57),173.0444(100)	4-*O*-caffeoylquinic acid	√
**20**	18.93		369.08279	0.2	$C_{16}H_{17}O_{10}$	-	isomer of fraxin	
**21**[a]	19.46	292, 336	429.10388	0.1	$C_{18}H_{21}O_{12}$	MS^2[429] 221.04532 (100)	eleutheroside B_1	√
**22** [a]	19.67	–	369.0823[c]	0.03	$C_{16}H_{17}O_{10}$	MS^2[369]207.0294(100)	Fraxin	√
**23**	19.91		595.16693	-0.2	$C_{27}H_{31}O_{15}$	MS^2[595] 355.08160 (100), 385.09222 (80), 475.12350 (90)	Unknown	√
**24** [a]	20.05		177.01955	1.2	$C_9H_5O_4$	MS^2[177] 133.02963 (100)	Eculetin	
**25**	20.54	239, 296, 323	349.06009	-1.0	$C_{13}H_{17}O_9S$	MS^2[349] 241.00212 (100)	sulfur-containing derivatives	√
**26**[a]	20.61	217,323	179.0332	-3.2	$C_9H_7O_4$	MS^2[179]135.0451 (100)	caffeic acid	√
**27**[a]	21.62	216, 327	335.07703	-1.6	$C_{16}H_{15}O_8$	MS^2[335]291.0867(24), 273.0762 (4), 247. 0971(9),229.0867(4),179.0347(100),135.0451(13)	3-*O*-caffeoylshikimic acid	
**28**[a]	22.15	216,327	335.07703	-0.8	$C_{16}H_{15}O_8$	MS^2[335]291.0867(27),247.0971(4),229.0866(2),179.0347(100), 161.0423(87),135.0451(32)	5-*O*-caffeoylshikimic acid	
**29**	22.54		337.09305	-0.5	$C_{16}H_{17}O_8$	MS^2[337] 191.05602 (100)	dihydro caffeoylquinic acid	

**30**	23.05		305.10629	-0.4	$C_{13}H_{21}O_6S$	MS^2[305] 96.96041(100)	sulfur-containing derivatives	
**31** [a]	23.38		207.02998	0.4	$C_{10}H_7O_5$	MS^2[207] 192.00616 (100)	fraxidin	√
**32** [a]	23.76	217, 327	335.07693	0.5	$C_{16}H_{15}O_8$	MS^2[335]291.0862(1),179.0346(100),161.0242(3),135.0450(23)	4-*O*-caffeoylshikimic acid	√
**33** [a]	23.94	–	431.19229	0	$C_{20}H_{31}O_{10}$	MS^2[431]385.1846(100)	drovomifoliol-*O*-*B*-D-glucopyranoside	
**34**	24.82		565.25012	-0.1	$C_{25}H_{41}O_{14}$	MS^2[565] 519.24365(100)	Unknown	
**35** [a]	25.21	–	433.20779	-0.3	$C_{20}H_{33}O_{10}$	MS^2[433]387.20166 (100)	dihydrovomifoliol-*O*-*B*-D-glucopyranoside	√
**36** [a]	25.28		433.11398	-0.1	$C_{21}H_{21}O_{10}$	MS^2[433] 343.08167 (20), 313.07132 (100)	(2R/2*S*)-naringenin-6-C-*B*-D-glucopyranoside	√
**37** [a]	25.87	228,290	565.1548[c]	-0.7	$C_{26}H_{29}O_{14}$	MS^2[565]547.1445(4),529.1342(1),397.0919(7),343.0813(36),313.0710(100)	(2*R/2S*)-naringenin-6-C-*B*-D-glucopyranosyl-(6→1)-apiose	√
**38** [a]	26.00	–	471.18719	-0.4	$C_{22}H_{31}O_{11}$	MS^2[471]425.1804(100)	sarcaglaboside G	√
**39** [a]	26.31	234,291	433.11401	0	$C_{21}H_{21}O_{10}$	MS^2[433]415.1019(4),397.0910(1),343.0807(18),313.0705(100)	(2*R/2S*)-naringenin-6-C-*B*-D-glucopyranoside	√
**40**	26.38		363.07556	0.1	$C_{14}H_{19}O_9S$	MS^2[363] 241.00200, 180.98112	Unknown	
**41** [a]	26.90	233,289	303.05078	1.3	$C_{15}H_{11}O_7$	MS^2[303]285.0396(100)	taxifolin	√
**42** [a]	27.08	228, 290	565.1547 [c]	-0.8	$C_{26}H_{29}O_{14}$	MS^2[565]547.1447(4),529.1338(1),397.0919(7),343.0814(36),313.0710(100)	(2R/2*S*)-naringenin-6-C-*B*-D-glucopyranosyl- (6→1)-apiose	√
**43** [c]	27.39		411.13297	-0.2	$C_{16}H_{27}O_{10}S$	MS^2[411] 241.00195 (100), 241.00195 (20)	sulfur-containing derivatives	√
**44** [a]	27.55	249,299	339.07199	-0.5	$C_{15}H_{15}O_9$	MS^2[339]205.0128 (19),193.0129	4,5-dihydroxy-7-rhamnyl-2H-chr	√

						(100) ,192.00629 (80)	omen-2-one	
**45** [a]	27.73	341	221.0454 [c]	-1.6	$C_{11}H_9O_5$	MS^2[221]206.0207(100)	isofraxidin	√
**46**	28.15		567.20825	-0.1	$C_{27}H_{35}O_{13}$	MS^2[567] 521.20190, 359.14948, 341.13864, 329.13867	Unknown	
**47**	28.22	–	473.20300	0.3	$C_{22}H_{33}O_{11}$	MS^2[473]427.1960 (100)	sarcaglaboside H	√
**48**	28.88		363.07553	0	$C_{14}H_{19}O_9S$	MS^2[363]241.00253(100), 180.98152 (11)	sulfur-containing derivatives	
**49**	29.22		603.22931	-0.2	$C_{27}H_{39}O_{15}$	MS^2[603] 557.22296 (100)	Unknown	
**50**	29.37		515.11945	-0.1	$C_{25}H_{23}O_{12}$	MS^2[515] 353.08719 (100)	di-caffeoylquinic acid	√
**51**	29.72		719.16095	-1.1	$C_{36}H_{31}O_{16}$	MS^2[719] 359.07690 (100)	rosmarinic acid dimers	√
**52** [a]	29.76	289	449.1089 [c]	-0.1	$C_{21}H_{21}O_{11}$	MS^2[449]303.0501(100),285.0396(78)	neoastilbin	√
**53** [a]	29.96		515.11957	0.2	$C_{25}H_{23}O_{12}$	MS^2[515] 353.08713	3, 5-caffeoylquinic acid	
**54** [a]	30.65	289	449.1083 [c]	-1.4	$C_{21}H_{21}O_{11}$	MS^2[449]303.0501(100),285.0396(86)	astilbin	√
**55** [a]	30.89	280	469.17154	0	$C_{22}H_{29}O_{11}$	MS^2[469] 423.16531 (100)	Chloranoside A	√
**56** [a]	31.13	218,328	521.13000	-0.1	$C_{24}H_{25}O_{13}$	MS^2[521]359.0749(100)	Rosmarinic acid-4-*O*-*β*-D-glucoside	√
**57** [a]	31.58	254,250	477.06741	-0.1	$C_{21}H_{17}O_{13}$	MS^2[477] 301.03491 (100)	quercetin-3-*O*-*β*-D-glucuronide	√
**58**	31.80		567.20825	-0.1	$C_{27}H_{35}O_{13}$	MS^2[567] 521.20184	Unknown	
**59**	32.24	233,281	597.1608 [c]	-0.6	$C_{30}H_{29}O_{13}$	MS^2[597]451.1010 (100)	Glabraoside A	
**60** [a]	33.24	248,329	515.11945			MS^2[515] 353.08716	4,5-dicaffeoylquinic acid	
**61** [a]	33.61		435.12973	0.1	$C_{21}H_{23}O_{10}$	–	phlorizin	
**62** [a]	32.82	290	449.1077 [c]	-0.4	$C_{21}H_{21}O_{11}$	MS^2[449] 303.0499(100),285.0395(77)	neoisoastilbin	√
**63** [a]	33.45	294	449.1078 [c]	-0.1	$C_{21}H_{21}O_{11}$	MS^2[449]303.0501(100),285.0396(72)	isoastilbin	√
**64** [a]	33.82	328	359.07715	-0.3	$C_{18}H_{15}O_8$	MS^2[359]223.0229(12),197.0438(29), 179.0334(26), 161.0230(100)	rosmarinic acid	√

**65**	34.57		453.17981	-0.4	$C_{19}H_{33}O_{10}S$	MS^2[453] 256.99686 (10),241.00192 (100), 180.98109 (20)	sulfur-containing derivatives	
**66**	34.81	294	449.14850	-0.3	$C_{19}H_{29}O_{10}S$	MS^2[449] 241.00192 (100), 180.98108 (72)	sulfur-containing derivatives	
**67** [a]	35.26		447.09308	-0.5	$C_{21}H_{19}O_{11}$	MS^2[447] 301.03488 (100), 300.02722 (30)	quercitrin	√
**68**	35.36		461.07245	-0.2	$C_{21}H_{17}O_{12}$	MS^2[461] 285.03995 (100)	Kaempferol-3-*O*-*β*-D-glucuronide	√
**69**	35.78	_	587.23438	-0.3	$C_{27}H_{39}O_{14}$	MS^2[587] 541.22772 (100)	sarcaglaboside E	
**70**	36.21		289.11136	-0.5	$C_{13}H_{21}O_5S$	MS^2[289] 96.96040 (100)	sulfur-containing derivatives	√
**71**	36.38		451.16412	-0.5	$C_{19}H_{31}O_{10}S$	MS^2[451] 241.00195 (100)	sulfur-containing derivatives	
**72**	36.83		279.12405	0.9	$C_{15}H_{19}O_5$	MS^2[279] 235.13376(80), 207.13893 (28), 191.14409 (100)	sesquiterpenoid lactone	√
**73** [a]	36.87	263	423.16580	-0.6	$C_{21}H_{27}O_9$	MS^2[423] 279.12341 (100)	Sarcaboside A	√
**74**	37.61	228	279.12369	-0.4	$C_{15}H_{19}O_5$	MS^2[279] 235.13371 (100), 235.13371 (50), 191.14400 (100)	sesquiterpenoid lactone	√
**75**	37.93		343.08200	-1.0	$C_{18}H_{15}O_7$	MS^2[343]181.05045(10), 161.02429(100)	unknown	√
**76**	38.75	263	279.12360	-1.9	$C_{15}H_{19}O_5$	MS^2[279] 235.13359 (100)	sesquiterpene lactone	√
**77**	39.43		263.12875	-0.5	$C_{15}H_{19}O_4$	MS^2[263] 219.13875 (30), 173.09708 (20), 153.09206 (100)	(-)-istanbulin A	√
**78**	40.07		293.10297	-0.3	$C_{15}H_{17}O_6$	MS^2[293] 249.11293 (100), 205.12325 (86)	sesquiterpene lactone	√

**79**	40.25		539.22839	-0.5	$C_{30}H_{35}O_9$	MS^2[539] 495.23816(100)	unknown	
**80**	40.71		259.09741	-0.7	$C_{15}H_{15}O_4$	MS^2[259]215.10747 (100)	sesquiterpene lactone	√
**81**	41.07	_	453.1762	-1.0	$C_{22}H_{29}O_{10}$	MS^2[453] 407.17032 (100)	sarcaglaboside B	
**82** [a]	42.19		205.05069	0.3	$C_{11}H_9O_4$	MS^2[205] 161.02435 (100)	vinyl caffeate	√
**83**	43.26	–	455.1916 [d]	-1.5	$C_{22}H_{31}O_{10}$	MS^2[455]409.1857(100)	sarcaglaboside C	√
**84**	42.73		293.10291	-0.5	$C_{15}H_{17}O_6$	249.11282(100), 231.10236(70), 205.12317 (76)	sesquiterpene lactone	√
**85**	43.26	–	455.1918 [d]	-1.0	$C_{22}H_{31}O_{10}$	MS^2[455]409.1854 (100)	sarcaglaboside A	
**86** [a]	43.26	232,270	261.11316	-0.3	$C_{15}H_{17}O_4$	MS^2[261]243.1015(18),217.1224(100),199.1120 (30), 173.1329 (20), 140.0111 (46)	8β,9α-dihydroxylindan-(5),7(1)-ieb-8α, 12-olide	√
**87**	44.65		493.22910	0.1	$C_{22}H_{37}O_{12}$	447.22269 (100)	unknown	
**88** [a]	44.45		299.09207	-1.4	$C_{17}H_{15}O_5$	MS^2[299]284.06851(100), 269.04520 (13)	5-hydroxy-7,8-dimethoxy-flavanone	√
**89** [a]	47.46		261.11313	-0.4	$C_{15}H_{17}O_4$	MS^2[261] 217.12323 (100), 217.12323 (22)	chloranthalactone E	√

[a] Compared with reference standards

√Compounds absorbed into blood as original forms after oral administration of *S.glabra* extract

–Their UV spectra have not been properly observed due to low intensity, overlapped or lack reference standards
