# Supplementary material for: Systematic Analysis of Absorbed Anti-Inflammatory Constituents and Metabolites of Sarcandra glabra in Rat Plasma Using Ultra-High-Pressure Liquid Chromatography Coupled with Linear Trap Quadrupole Orbitrap Mass Spectrometry
Source: PLoS One. 2016 Mar 14;11(3):e0150063. doi: 10.1371/journal.pone.0150063 (PMC4790918; doi:10.1371/journal.pone.0150063)
Supplement: S3 Table — (PDF) [file pone.0150063.s005.pdf]

**S3 Table. Predicted metabolites of 18, 21, 45, 54, 56, 57 and 64 in rat plasma after oral administration**

| **Expected $t_R$(min)** [a] | **[M-H]$^-$** | **Predicted product ions** | **biotransformation** | **Change in MW (Da)** | **Predicted matabolites** |
|---|---|---|---|---|---|
| Predicted metabolites of 5-*O*-caffeoylquinic acid (**18**) | | | | | |
| 12.4 | 353.1 | 191.0 | Position isomer | 0 | 3-*O*-caffeoylquinic acid (**12**) |
| 17.9 | 353.1 | 191.0 | Parent | 0 | 5-*O*-caffeoylquinic acid (**18**) |
| 18.5 | 353.1 | 173.0 | Position isomer | 0 | 4-*O*-caffeoylquinic acid (**19**) |
| 20.6 | 179.1 | 135.1 | Hydrolysis | −174 | caffeic acid (**26**) |
| 3.6 | 191.1 | 173.1,127.2 | Hydrolysis | −162 | quinic acid (**1**) |
| – | 355.1 | 179.1 | Hydrolysis+ Glucuronidation | −174+176 | caffeic acid glucuronide |
| – | 259.1 | 179.1 | Hydrolysis+ Sulfation | −174+80 | caffeic acid-O-sulfate |
| – | 243.1 | 163.1 | Hydrolysis+Deoxy+Sulfation | −174−16+80 | (*E*)-3-(4-(sulfooxy)phenyl)acrylic acid |
| – | 435.1 | 179.1 | Hydrolysis+Sulfation+Glucuronidation | −174+80+176 | caffeic acid-O-glucuronide-O- sulfate |
| – | 273.1 | 193.0 | Hydrolysis+ Methylation+Sulfation | −174+14+80 | methylated caffeic acid-O-sulfate |
| – | 193.0 | 178.0 | Hydrolysis+ Methylation | −174+14 | ferulic acid / isoferulic acid |
| – | 223.1 | 208.1 | Hydrolysis+ 2×Methylation | −174+28 | Sinapic acid |
| 27.0 | 163.0 | 119.0 | Hydrolysis+Deoxy | −174−16 | *p*-coumaric acid (**98**) |

| | | | | | |
|---|---|---|---|---|---|
| | 165.0 | 121.0 | Hydrolysis+Deoxy+ Hydrogenation | −174−16+2 | 3-Hydroxyphenylpropionic acid |
| − | 355.0 | 311, 179 | Hydrogenation | +2 | Dihydrocaffeoylquinic acid |
| − | 367.0 | 191.1 | Methylation | +14 | methylated caffeoylquinic acid |
| − | 545.1 | 179.1 | Methylation + Cysteinylglycine | +14+178 | methylation cysteinylglycine conjugation |
| Predicted metabolites of isofraxidin (**45**) | | | | | |
| 27.7 | 221.0 | 206.0 | Parent | 0 | isofraxidin (**45**) |
| − | 301.0 | 221.1 | Sulfation | +80 | isofraxidin -O-sulfate |
| − | 191.1 | 163.0 | Deoxymethyl | −30 | Deoxymethyl conjugation |
| − | 161.1 | 133.0 | 2×Deoxymethyl | −60 | 2×Deoxymethyl conjugation |
| 19.5 | 429.1[b] | 221.0 | Glucosylation | +162 | eleutheroside $B_1$ |
| − | 235.1 | 220.0 | Methylation | +14 | methylated isofraxidin |
| − | 397.1 | 221.0 | Glucuronidation | +176 | isofraxidin-O-glucuronide |
| Predicted metabolites of rosmarinic acid (**64**) | | | | | |
| 33.8 | 359.1 | 161.0 | Parent | 0 | rosmarinic acid |
| − | 453.1 | 373.1 | Methylation+Sulfation | +14+80 | methylated rosmarinic acid -O-sulfate |
| − | 535.1 | 359.1 | Glucuronidation | +176 | rosmarinic acid-O-glucuronide |
| 31.1 | 521.1 | 359.1 | Glucosylation | +162 | rosmarinic acid-4-*O*-*β*-D-glucoside |

| | | | | | |
|---|---|---|---|---|---|
| − | 439.1 | 359.1 | Sulfation | +80 | rosmarinic acid-O-sulfate |
| − | 549.1 | 373.1 | Methylation+ Glucuronidation | +14+176 | methylated rosmarinic acid -O-glucuronide |
| − | 563.1 | 359.1 | Dimethylation+ Glucuronidation | +28+176 | dimethylated rosmarinic acid-O- glucuronide |
| − | 373.1 | 161.1 | Methylation | +14 | methylated rosmarinic acid |
| 21.1 | 179.1 | 135.1 | Hydrolysis | −180 | caffeic acid (**26**) |
| − | 259.1 | 179.1 | Hydrolysis+Sulfation | −180+80 | caffeic acid-O-sulfate |
| 27.0 | 163.1 | 119.1 | Hydrolysis+Deoxy | −180−16 | *p*-coumaric acid (**98**) |
| − | 243.1 | 163.1 | Hydrolysis+Deoxy+Sulfation | −180−16+80 | coumaric acid sulfate |
| − | 339.1 | 163.1 | Hydrolysis+Deoxy+Glucuronidation | −180−16+176 | coumaric acid-O-glucuronide |
| − | 193.1 | 178.0 | Hydrolysis+ Methylation | −180+14 | methylated caffeic acid |
| − | 273.1 | 193.1 | Hydrolysis+ Methylation+ Sulfation | −180+14+80 | methylated caffeic acid-O-sulfate |
| − | 165.1 | 121.1 | Hydrolysis+Deoxy+Hydrogenation | −180−16+2 | 3-(3-hydroxyphenyl)propanoic acid |
| − | 261.1 | 181.1 | Hydrolysis+Hydrogenation+ Sulfation | −180+2+80 | dihydrocaffeic acid-O-sulfate |

Predicted metabolites of astilbin (**54**)

| | | | | | |
|---|---|---|---|---|---|
| 30.0 | 449.1 | 303.1 | Configurational isomer | 0 | neoastilbin (**52**) |
| 30.7 | 449.1 | 303.1 | Parent | 0 | astilbin (**54**) |
| 32.8 | 449.1 | 303.1 | Configurational isomer | 0 | neoisoastilbin (**62**) |
| 33.5 | 449.1 | 303.1 | Configurational isomer | 0 | isoastilbin (**63**) |
| – | 625.1 | 449.1 | Glucuronidation | +176 | astilbin O-glucuronide |
| – | 529.1 | 449.1 | Sulfation | +80 | astilbin O-sulfate |
| – | 463.1 | 299.1 | Methylation | +14 | 3'-O-methylated astilbin |
| – | 639.1 | 463.1 | Methylation+ Glucuronidation | +14+176 | methylated astilbin O-glucuronide |
| – | 543.1 | 463.1 | Methylation+ Sulfation | +14+80 | methylated astilbin-O-sulfate |
| – | 303.1 | 285.0 | Hydrolysis | -146 | taxifolin |
| Predicted metabolites of rosmarinic acid-4-O-β-D-glucoside (**56**) | | | | | |
| 31.1 | 521.1 | 359.1 | Parent | 0 | rosmarinic acid-4-O-β-D-glucoside (**56**) |
| 33.8 | 359.1 | 161.0 | Hydrolysis | -162 | rosmarinic acid (**64**) |
| – | 535.1 | 359.1 | Methylation | +14 | Methylated rosmarinic acid-4-O-β-D- glucoside |

| | | | | | |
|---|---|---|---|---|---|
| − | 697.1 | 521.1 | Glucuronidation | +176 | rosmarinic acid-4-O-β-D-glucoside-O- glucuronide |
| − | 615.1 | 535.2, 373.2 | Methylation+Sulfation | +14+80 | methylated rosmarinic acid-4-O-β-D- glucoside-O-sulfate |
| − | 601.1 | 521.1 | Sulfation | +80 | rosmarinic acid-4-O-β-D-glucoside-O- sulfate |
| Predicted metabolites of eleutheroside $B_1$ (**21**) | | | | | |
| 19.5 | 429.1[b] | 221.1 | Parent | 0 | eleutheroside $B_1$ (**21**) |
| 27.7 | 221.1 | 206.0 | Hydrolysis | -162 | isofraxidin (**45**) |
| − | 509.1 | 429.1, 221.1 | Sulfation | +80 | eleutheroside $B_1$-O-sulfate |
| − | 397.1 | 221.1 | Hydrolysis+Glucuronidation | -162+176 | isofraxidin - O-glucuronide (**101)** |
| − | 369.1 | 207.1 | Demethylation | −14 | fraxin |
| − | 355.1 | 207.1 | Demethylation×2 | −28 | Demethylation fraxin |
| − | 301.1 | 221.1 | Hydrolysis+Sulfation | -162+80 | isofraxidin-O-sulfate (**100**) |
| − | 353.1 | 191.1 | Deoxymethylation | -30 | -hydroxymethyl |
| − | 323.1 | 161.1 | Deoxymethylation×2 | -60 | -dihydroxymethyl |
| − | 207.1 | 192.0 | Hydrolysis+ Demethylation | -162-14 | 5,6-dihydroxyl-7-methoxycoumarin |
| Predicted metabolites of quercetin-3-*O*-*β*-D-glucuronide (**57**) | | | | | |
| 31.9 | 477.1 | 301.1 | Parent | 0 | quercetin-3-*O*-*β*-D-glucuronide (**57**) |

| | | | | | |
|---|---|---|---|---|---|
| – | 301.1 | 179.1 | Hydrolysis | -176 | quercetin |
| – | 381.1 | 301.1 | Hydrolysis+Sulfation | -176+80 | quercetin-O-sulfate |
| – | 653.2 | 477.1, 301.1 | Glucuronidation | +176 | quercetin-3-*O*-*β*-D-glucuronide O-glucuronide |
| – | 667.2 | 491.1, 315.1 | Methylation+Glucuronidation | +14+176 | methyl quercetin-3-*O*-*β*-D-glucuronide O- glucuronide |
| – | 639.2 | 477.1,301.1 | Glucosylation | +162 | quercetin-3-*O*-*β*-D-glucuronide-O-glucoside |
| – | 625.2 | 463.1,301.1 | Glucosylation+ Glucosylation | +324 | quercetin-O-glucoside-O-glucoside |
| – | 463.1 | 301.1 | Hydrolysis+ Glucosylation | -176+162 | quercetin-O-glucoside |
| – | 543.1 | 463,381,301 | Hydrolysis+ Glucosylation+ Sulfation | -176+162+80 | quercetin-O-glucoside-O-sulfate |
| – | 557.1 | 477, 381,301 | Sulfation | +80 | quercetin-3-*O*-*β*-D-glucuronide-O-sulfate |
| – | 491.1 | 315.1 | Methylation | +14 | methyl quercetin-3-*O*-*β*-D-glucuronide |

[a]Refer to those absorbed constituents or metabolites with known analytical retention time.

[b]Detected as $[M+HCOOH-H]^-$
